# Structural and Functional Co‐Adaptation of Plants of the Genus *Lysimachia* L. (Primulaceae) and Pollinating Insects of the Genus *Macropis* Panzer (Hymenoptera, Melittidae)

**DOI:** 10.1002/ece3.72544

**Published:** 2025-11-26

**Authors:** Vladimir G. Radchenko, Mykola G. Chausov, Artur F. Likhanov, Hanna Yu. Honchar, Denis Michez

**Affiliations:** ^1^ Institute for Evolutionary Ecology of the National Academy of Sciences of Ukraine Kyiv Ukraine; ^2^ The National University of Life and Environmental Sciences of Ukraine Kyiv Ukraine; ^3^ Laboratory of Zoology, Research Institute for Biosciences University of Mons Mons Belgium

**Keywords:** bees, behaviour, floral oil, morphology, polysaccharides, trichomes

## Abstract

The pollination system ‘oil‐collecting bees *Macropis* and oil‐producing plants *Lysimachia*’, probably originated a long time ago. Although much is already known about the biology and ecology of this fascinating interaction, we reveal here features of the structural elements of the secretory system of the *Lysimachia* flowers and the foraging behaviour of *Macropis* bees. A model describing the process of collecting flower oil by bees based on the laws of mechanics is presented. The model considers the histochemical and morphological features of the secretory apparatus in *Lysimachia* flowers, as well as the behaviour and micromorphological structure of the oil‐ and pollen‐collecting apparatus of the bee. All these structures seem to be adapted for collecting flower oil and other metabolic products, demonstrating a process of co‐adaptation. It has been clarified for the first time that *Macropis* females do not merely collect pure floral oil through capillary absorption or by mopping up oil from trichomes on the flower stamens and petals via specialised pubescence on the fore‐ and mid‐tibia and tarsi of bees, as was previously thought. Instead, they collect entire oil‐filled trichome heads by breaking them off from the trichome stems. A neck (intermediate) cell has been described in the oil‐secreting capitate trichomes of *Lysimachia nummularia* L. The dominance of pectin and hemicellulose in its cell walls, combined with high beta‐galactosidase activity during trichome maturation, creates conditions for the formation of a microabscission zone. This zone facilitates the detachment of the oil‐containing capitate when contact occurs with pollinating bees of the genus *Macropis*.

## Graphical abstract

The co‐adaptation of bees from the genus Macropis and Lysimachia plants has been studied in detail. Research shows that bees collect not just pure liquid oil from flower petals but tear off trichome heads filled with oil, transferring them to their nests. The morphological and physiological mechanisms that facilitate the process by which bees gather trichome heads have been established for the first time. A model describing the process of flower oil collection by bees, based on the laws of mechanics, is presented.

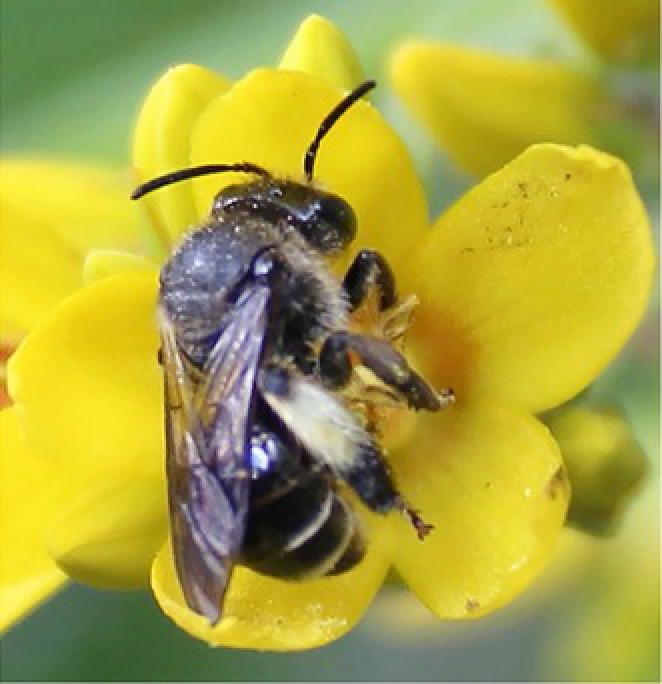

## 1 Introduction

The coevolution of plants and insects is primarily driven by the resources insects forage for and the reproductive or survival needs of plants on the other side (Futuyma and Agrawal 2009). Recent evidence suggests that insect pollination of plants first evolved in gymnosperms well before angiosperms appeared, and was performed by various insect groups (Peris et al. 2025) that were probably not specialised plant pollinators. The mutualistic ‘plants—pollinators’ system developed later over tens of millions of years, achieving great success for two clades: the bees (Anthophila) and the Angiosperms (Cardinal and Danforth 2013). However, it remains unclear how much the diversification of bees aligned with that of flowering plants (Van der Niet and Johnson 2012; Cappellari et al. 2013). Furthermore, recent palaeontological studies and molecular analyses challenge the idea of an inextricable link between angiosperms and insect pollinators during their early diversification stages (Van der Kooi and Ollerton 2020). Discrepancies in the emergence dates of major angiosperm and insect lineages have been recognised (Asar et al. 2022). The selection of bees and flowering plants during their coevolution led to a tremendous diversification in terms of the number of species, their morphological structures and behaviour (Radchenko and Pesenko 1994; Ollerton 1999; Bronstein et al. 2006; Van der Niet and Johnson 2012; Cardinal and Danforth 2013). Today, the mutualistic system includes over 20,000 bee species (Ascher and Pickering 2024) and approximately 300,000 entomophilous plant species, representing an average of 87.5% (with variations across different climatic zones from 78% to 94%) of around 350,000 known species of flowering plants (Ollerton et al. 2011; Tong et al. 2023).

Plants display a range of traits associated with attracting a specific group of pollinators, known as the pollination syndrome (Hermann and Kuhlemeier 2011). These traits include flower characteristics, such as the colour and size of the corolla, as well as rewards like pollen, nectar, perfume, or oil. Plant traits can also be biochemical, like the chemical composition of pollen (e.g., protein/lipid ratio) and nectar (e.g., sugar concentration) or the floral scent (Dobson 1987; Lunau 1996; Dobson and Bergström 2000; Roulston et al. 2000; Schiestl and Peakall 2005; Dötterl and Vereecken 2010; Dötterl et al. 2005; Dötterl and Schäffler 2007; Burger et al. 2012; Vanderplanck et al. 2017; Sasidharan et al. 2023).

At the same time, bees show physiological adaptations necessary to digest nectar, pollen or oil (Pesenko and Radchenko 1993; Radchenko et al. 1993; Eckhardt et al. 2014; Vaudo et al. 2015, 2024; Rivest and Forrest 2020). Phenological adaptations are shown in the synchronisation of blooming and flight (Pesenko et al. 1980; Bartomeus et al. 2011; Edge et al. 2012; Kehrberger and Holzschuh 2019). Ethological adaptations are realised through special behaviour patterns for effectively collecting and transferring pollen, nectar, flower oil, resins, and other plant and mineral materials.

Most of the studied bees are polylectic and do not have a restricted relationship with specific food plants (Radchenko and Pesenko 1994; Lechantre et al. 2021). However, many bee species share similar morphological features, including *special anatomical* structures that assist them in collecting and transporting pollen to their nests (Vogel 1971, 1974; Roberts and Vallespir 1978; Thorp 1979, 2000; Neff and Simpson 1981, 2017; Fenster et al. 2004; Pauw et al. 2017; Portman and Tepedino 2017).

Generally, mutualistic relationships between plants and pollinators are not linked to specific co‐adaptation. Instead, plants have a diverse range of pollinators, which also visit many different plants; that is, they are polylectic. About one‐third of European bee species with known trophic links to plants have limited trophic relationships (Radchenko and Pesenko 1994; Bogusch et al. 2020). These bee species with a limited range of food plants show the most remarkable adaptations to their food plants.

The most apparent signs of mutualism, along with the possibly co‐evolution of traits related to plant pollination and bee foraging, are typically seen only in highly specialised systems with very narrow trophic links, limited to plants of a single genus or even a single species, and their specialised pollinators, which also belong to the same genus or even a single species.

Among the co‐adaptations of plants and pollinators, it should be noted that flower depth and pollinator proboscis length are generally correlated (Stang et al. 2009; Pyke 2016; Klumpers et al. 2019). For example, the detailed functional morphology of the head and proboscis of *Andrena lonicerae* Tadauchi & Hirashima, 1988, is specifically adapted to the morphology and nectar production of *Lonicera gracilipes* Miq. flowers (Shimizu et al. 2014). Some plants across various parts of their range can vary greatly, particularly in flower size, demonstrating clear adaptation to specific pollinators. Thus, the plant *Calceolaria polyrhiza* Cav., in one part of its range, has only one pollinator, *Chalepogenus caeruleus* (Friese, 1906) (tribe Tapinotaspidini), while in another part of its area, it is pollinated solely by the bee *Centris cineraria* Smith, 1854 (tribe Centridini). These two bee species differ markedly in their distribution ranges, body size, and behaviour on flowers. Respectively, the flower size of this plant varies according to the size of its pollinator in different parts of its range. Therefore, adaptive intraspecific flower differentiation is a response to geographic variation in pollinators. However, such adaptations are only expressed at the phenotypic level and cannot exemplify coevolution (Cosacov et al. 2014).

In narrow mutualistic ‘plant‐pollinator’ systems, the floral oil‐collecting bees warrant special attention, as this is a comparatively rare phenomenon. Currently, around 2000 plant species produce floral oil, and approximately 500 bee species have trophic relationships involving oil collection (Vogel 1971, 1974; Simpson et al. 1977; Simpson and Neff 1981; Buchmann and Buchmann 1981; Buchmann 1987; Rasmussen and Olesen 2000; Renner and Schaefer 2010; Martins et al. 2013; Carneiro and Machado 2023). The relationship between oil‐collecting bees and plant species that produce floral oil is mutualistic (Triponez et al. 2015). The comprehensive modern reviews of the information on the relationship between oil‐collecting bees and floral oil‐producing plants are provided in the works of Guimarães et al. (2021) and Carneiro and Machado (2023).

Oil‐producing plants and oil‐collecting bees are more diverse in the Neotropics (Renner and Schaefer 2010), and in this area most research has been conducted on such interactions between plants and bees (Guimarães et al. 2021). Many studies have also examined the collection of oil by certain bees in South Africa. In this context, among the most specialised systems with highly limited trophic links, researchers often focus on the mutual adaptation of bees of the neotropical genus *Centris* Fabricius, 1804 (Apidae), which collect floral oil from plants of the genus *Krameria* Loefling, 1758 (Krameriaceae), as well as some species of South African bees of the genus *Rediviva* Friese, 1911 (Melittidae), which collect oil from flowers of plants of the genus *Diascia* Link & Otto, 1820 (Scrophulariaceae) (Whitehead et al. 1984; Vogel 1984; Vogel and Michener 1985; Steiner and Whitehead 1988, 1990; Johnson and Steiner 2003; Kuhlmann and Hollens 2014; Martins et al. 2015; Pauw et al. 2017; Carneiro et al. 2019, 2024). The bees of the genus *Rediviva* utilise their extraordinarily long forelegs to collect floral oil from elaiophores located deep within the elongated flower spur of *Diascia*. In various *Rediviva* populations, there is a strong correlation between the average foreleg length and *Diascia* flower spur length, suggesting that plant‐driven selection may have influenced the evolution of these bees (Steiner and Whitehead 1990, 1991). However, recent studies show that even with such strong mutualistic relationships, there is no clear evidence of coevolution (Kahnt et al. 2019).

Only one system with oil‐producing plants and oil‐collecting bees is known in Europe; this involves the plants of the genus *Lysimachia* Linnaeus, 1753 and bees of the genus *Macropis* Panzer, 1809 (Michez and Patiny 2005).

In general, oil production evolved independently in multiple plant clades (Renner and Schaefer 2010). Accordingly, oil‐collecting behaviour appeared independently in different bee clades (Neff and Simpson 1981, 2017; Michez et al. 2012; Kuhlmann and Hollens 2014; Bossert et al. 2019; Policarová et al. 2019; Rasmussen et al. 2020). However, fossil evidence of the interactions between plants and pollinators is very rare (Ramírez et al. 2007). This makes it difficult to understand the evolution of their relationships. The genus *Lysimachia* has relatively young fossils from the Oligocene (Boucher et al. 2016), although the molecular age of its representatives falls approximately to the Late Eocene (Renner and Schaefer 2010). The *Macropis* clade occurred around this time. Since then, *Macropis* has apparently coevolved with *Lysimachia* (Michez et al. 2012). However, a probable ancestral form of the modern *Macropis* bees is known, namely *Palaeomacropis eocenicus* Michez and Nel 2007, whose legs' pubescence structure may indicate collecting plant oil existed even before the *Lysimachia* occurrence (Michez et al. 2007). Probably, it collected oil from other oil‐producing families, such as Malpighiaceae, which already existed in the early Eocene (Davis et al. 2002).

In the interactions between *Lysimachia* plants and *Macropis* bees, floral trichome elaiophores are essential. These capitate glandular trichomes (CGTs) accumulate oil. The specialisation of the *Macropis* bees in collecting the floral oil of *Lysimachia* plants, like the specialisations of many other oil‐collecting bees (e.g., *Ctenoplectra*, *Tetrapedia*, *Centris*, etc.), is primarily associated with the use of oil for lining underground nest cells, making them resistant to moisture and bacteria, and thus preserving the stored food and protecting the larvae feeding on it (Cane et al. 1983; Radchenko 1996; Alves‐dos‐Santos et al. 2006; Renner and Schaefer 2010; Kuhlmann 2014; Martins et al. 2015). It should be noted there that, according to Martins et al. (2015), the common ancestor of modern members of the genus *Centris*, prevalent in the deserts of Central America and xeric South America habitats, lost the oil‐collecting apparatus. This fact further supports the moisture‐protective role of floral oil in the cells of oil‐collecting bee species inhabiting mesophilic/wet biotopes.

Additionally, the stickiness of the oil applied to the hair helps pollen adhere to them, making it easier to transfer to the nest (Oliveira et al. 2022). *Macropis* bees also use *Lysimachia* oil not only as a building material but also as a component of the brood provision (Simpson and Neff 1981). The addition of oil to the provision possibly increases the nutritional value of feed since loosestrife pollen has a relatively low nutritional quality (Weiner et al. 2010).

Another very important function of adding oil to pollen food, according to Jerry Rozen (pers. com. in: Simpson and Neff 1981), with which we agree, is to prevent highly hygroscopic pollen from over‐moistening. This may be crucial for *Macropis* bees as they construct nests in mesophilic biotopes with relatively high soil moisture. They are constrained in these biotopes as it is the restricted biotope of their host plant, *Lysimachia*. Thus, the success of *Macropis* populations probably depends not only on the amount of pollen available in the flowers but also on the amount of oil collected and the efficiency of oil collection. In this regard, the plant secretory structures and their contents must be available to insects in sufficient amounts. In turn, insects need suitable morphological structures to collect pollen and oil from flowers, and plants require reliable cross‐pollination, which bees ensure by carrying pollen on their body surfaces.

The current understanding of how *Macropis* bees gather *Lysimachia* floral oil is mainly descriptive and based on researchers' observations of bee behaviour. It is widely accepted that when visiting *Lysimachia* flowers, *Macropis* bees directly collect pure oil, which is absorbed by the hair pads on their tibiae and tarsi of the fore and middle legs. Cane et al. (1983) believed that the oils of *Lysimachia ciliata* accumulate as shiny droplets at the tips of the trichome elaiophores, and females collect these oil droplets using specialised setaceous pads on the inner surfaces of their fore and middle tarsi. However, in reality, *Lysimachia* flowers have CGTs, and the oil is not located in the form of droplets on top of the trichomes, but is enclosed within the spherical trichome heads covered by cuticle (Simpson et al. 1983). Therefore, such a process of oil collection involves breaching the integrity of the surface of the integumentary walls of the secretory structures, which, according to various researchers, is achieved by breaking the outer walls of the elaiophores using pointed bristles on the front and middle legs of *Macropis* bees. Then, the oil is capillarily absorbed through specialised hair pads on the bees' legs (Vogel 1974; Michez et al. 2007, 2008). According to Roberts and Vallespir's data (1978), the hairs on the ventral tarsal surfaces of *Macropis nuda* forelegs are modified into an array of flattened, blade‐like teeth used for scraping oil.

Among other groups of oil‐collecting bees, specialised adaptive modifications in the structure of the leg pubescence should be noted in representatives of the genus *Centris*. Their oil‐collecting adaptations are in the shape of modified hairs on the basitarsus and several giant scapular hairs on the front and middle legs (Neff and Simpson 1981). Various types of hair for collecting oil were also noted on the legs of bees of the genera *Rediviva* (Kuhlmann and Hollens 2014) and *Tetrapedia* (Alves‐dos‐Santos Alves‐dos‐Santos et al. 2006), and in bees of the genus *Ctenoplectra*, inner spurs on the distal part of the hind tibia are modified into a comb‐like structure with dense teeth for combing out oil (Schaefer and Renner 2008).

In general, despite the different morphological structures of the oil‐collecting apparatus across various bee groups, oil is gathered either by wiping it from the surface of the trichomes, using the special pubescence on the tibiae and basitarsus of the fore and middle legs or dense setae on the ventral side of the matasome, or through capillary absorption of floral oil from trichomal or epithelial elaiophores using hair pads located on the fore and middle legs. In this process, the bees use stiff, spiky hairs on their legs near this pad to first rupture the cuticle covering the oil‐containing elaiophores.

A similar adaptation mechanism for collecting oil from *Lysimachia* flowers was assumed for *Macropis* by all authors studying these bees (Vogel 1974, 1986; Roberts and Vallespir 1978; Cane et al. 1983; Simpson et al. 1983; Rasmussen and Olesen 2000; Pekkarinen et al. 2003; Celary 2004; Michez and Patiny 2005; Schäffler and Dötterl 2011; Bassin et al. 2011; Homburger et al. 2025). However, these adaptations were described in very broad terms. The authors mentioned above did not clearly analyse the role of each morphological structure, leaving unclear the potential co‐adaptation between the oil secretory apparatus and the micromorphological peculiarities of the oil‐collecting structures on the bees' legs, as well as the bees' behaviour when collecting oil, for over 55 years.

The morphology and structural features of oil‐secreting trichomes have only been described for one species of the genus *Lysimachia*, *L. ciliata* L. (Simpson et al. 1983). In *Lysimachia ciliata* flowers, when describing the oil‐secreting CGTs, a two‐ or three‐celled stalk and an eight‐ or sixteen‐celled trichome head are typically distinguished (Simpson et al. 1983). In the structure of CGTs of other plant species, a specialised abscission zone is also distinguished at the junction of the head and stalk (Bergau et al. 2015). This zone can be represented by either a single intermediate cell (neck cell) or a group of cells (Hancock et al. 2024). The presence of an intermediate cell and a microabscission zone in *Lysimachia* CGTs has not been previously reported. However, based on studies of other plant species, it appears they must also form in *Lysimachia* trichomes.

It should also be noted that when studying the relationship between bees collecting floral oil and the plants that produce it, analysing the histochemical structure and biochemical composition of flowers and CGTs is essential. Such studies of oil‐producing plants in the genus *Lysimachia* mainly focus on the qualitative composition of metabolites in flowers and secretions in CGTs (Schäffler et al. 2012). However, the composition and structure of the polysaccharide complex in the cell walls of *Lysimachia* trichomes, which determine their strength and, consequently, influence the mechanics of oil collection, have not been studied at all.

Therefore, this study aims to develop a biomechanical model of floral oil collection by *Macropis* bees based on: (i) analysis of bee behaviour on flowers; (ii) investigation of specialised microstructures on bee legs and floral material collected by them; (iii) location and structure of CGTs; (iv) mapping of polysaccharide complexes within the cell walls of *Lysimachia* trichomes; (v) determination of cell‐specific localisation of β‐galactosidase activity associated with the modification of trichome cell walls.

## 2 Materials and Methods

**FIGURE 1:**
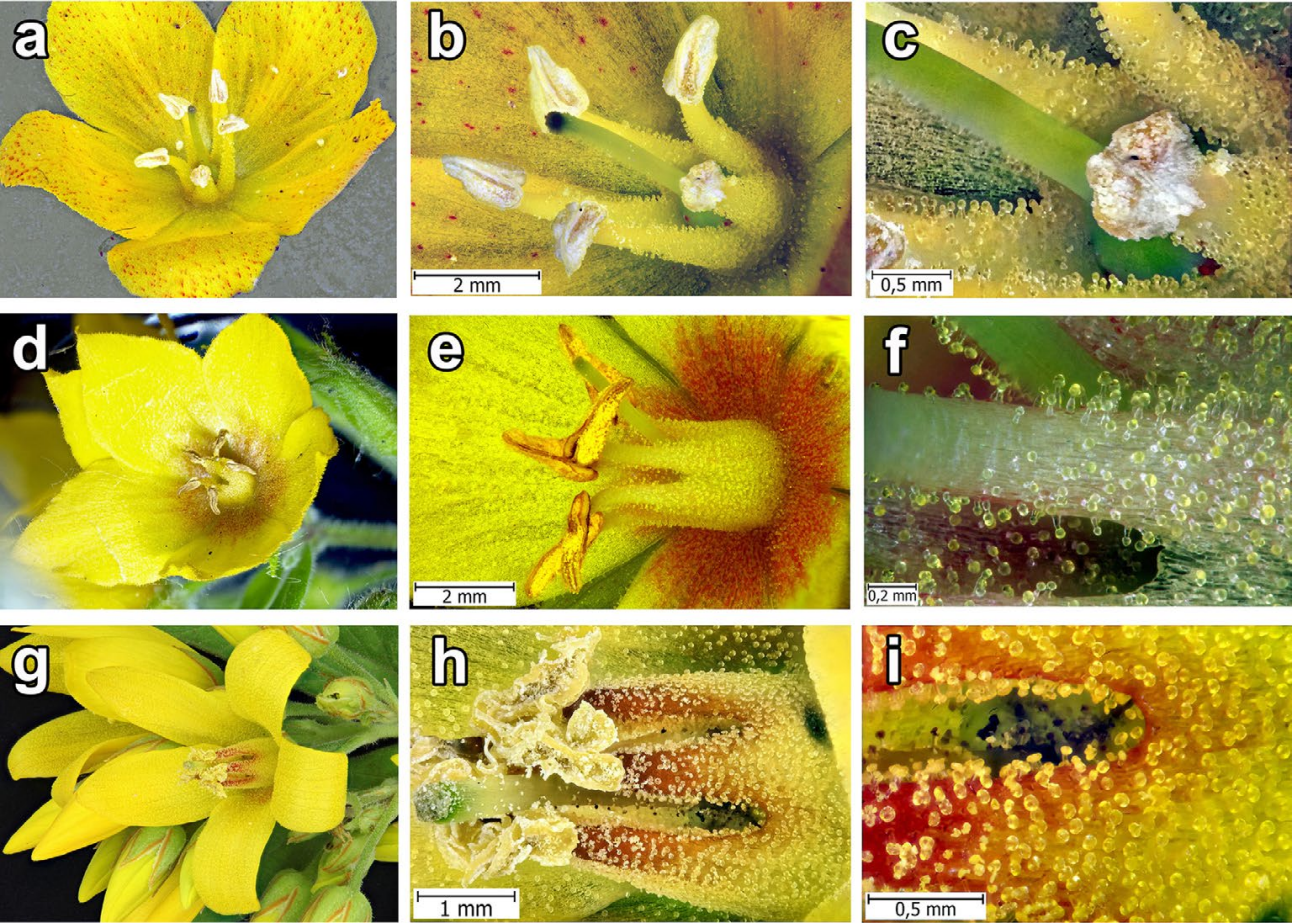
Flowers of *Lysimachia:* (a–c) *L. nummularia* L.; (d–f) *L. punctata* L.; (g–i) *L. vulgaris* L.

**FIGURE 2:**
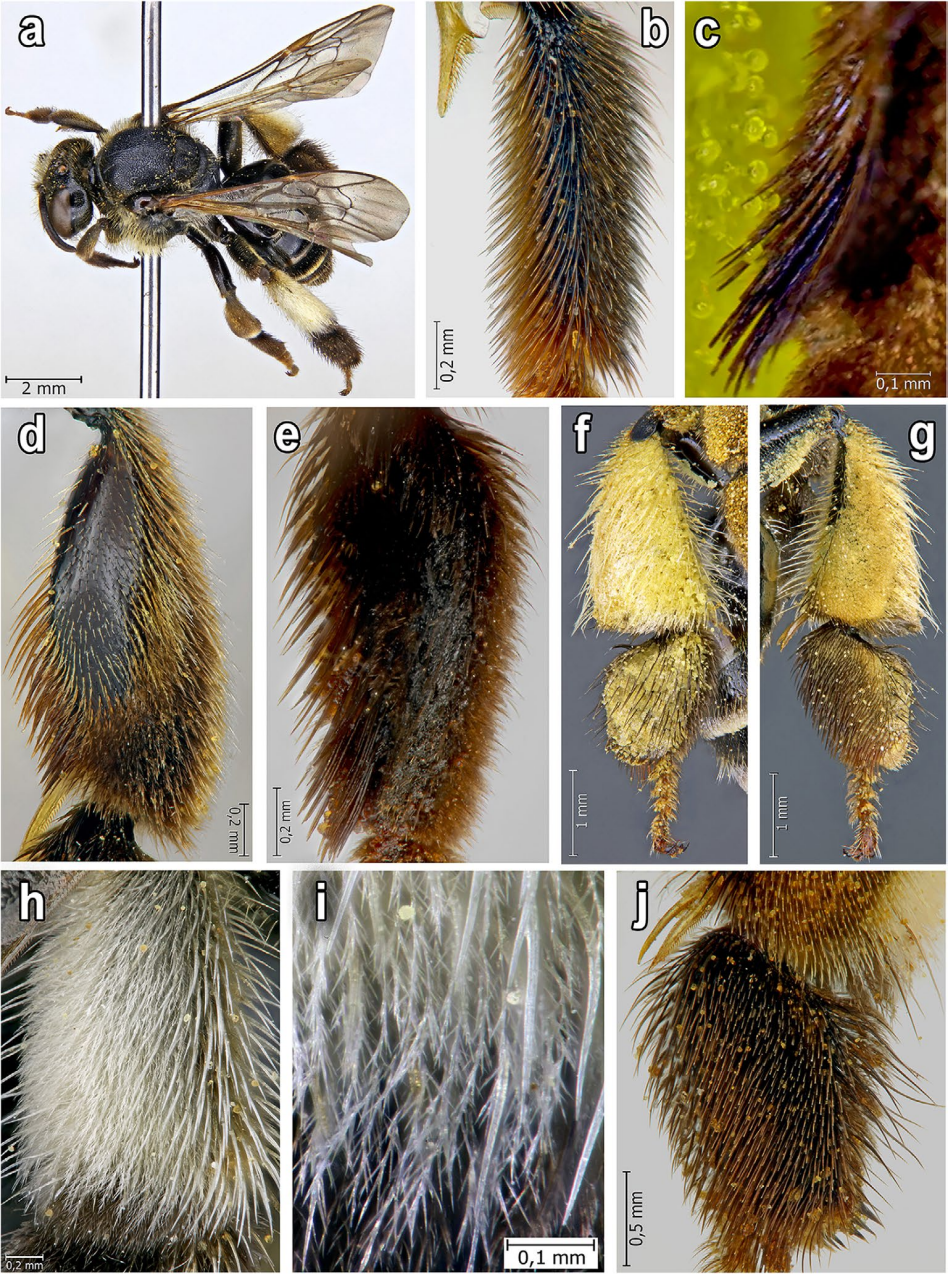
Female of *Macropis europaea* Warncke, 1973: (a) Habitus; (b) Forebasitarsus (frontal view); (c) Thick bristle‐like hairs on the forebasitarsus which are used for collecting oil‐containing trichome heads of *Lysimachia* flowers; (d) Inner side of the midtibia; (e) Inner side of the midbasitarsus; (f) Outer side of the hindleg; (g) Inner side of the hindleg; (h, i) Scopa on the metatibia with two types of hairs (bristle‐like and branched plumose hairs); (j) Inner side of metabasitarsus.

**FIGURE 3:**
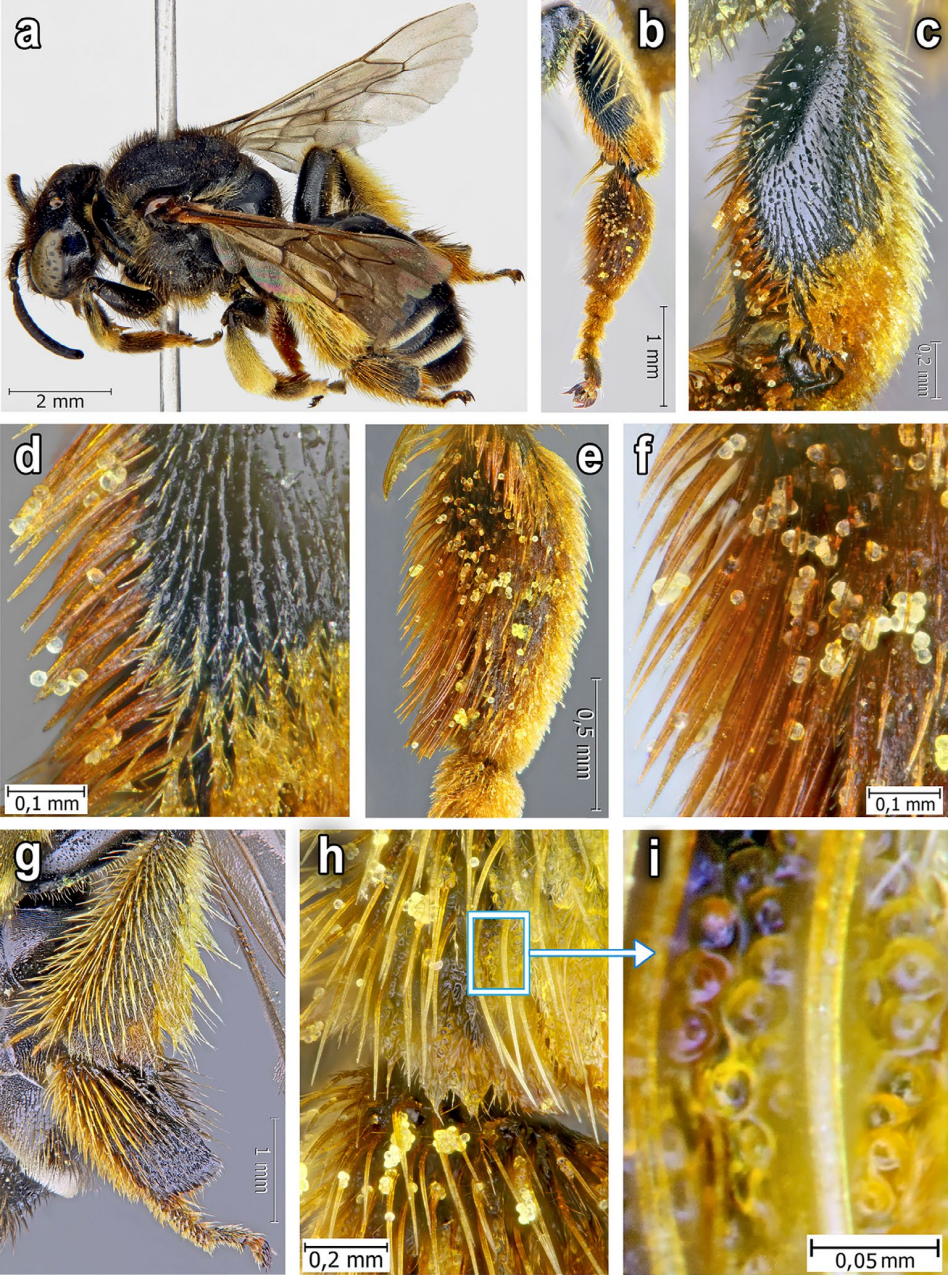
Female of *Macropis fulvipes* (Fabricius, 1805): (a) Habitus; (b) Middle leg (inner side); (c) Midtibia; (d) Apical half of midtibia; (e, f) Inner side of midbasitasus; (g) Hind leg (outer side); (h) Metatibia distal part and metabasitarsus basal part, carrying the floral oil; (i) Greatly magnified central part of metatibial scopa with leaked floral oil and preserved trichome heads filled with oil. Images b–f, h clearly show numerous oil‐filled, globular trichome heads located on top of and between the leg hairs of the bee.

The studies were carried out during 2015–2022 in the forest park plantations of Kyiv and its environs, in particular, on the island Muromets (50°30′22″ N 30°32′36″ E) and in «Feofaniya» Park, a monument of landscape art of national importance (50°20′20″ N 30°29′17″ E). *Lysimachia nummularia* L. (Figure 1a–c), *L. punctata* L. (Figure 1d–f) and *L. vulgaris* L. (Figure 1g–i), which are the most common species in the natural biotopes of the study area, and the bee species *Macropis europaea* Warncke, 1973 (Figure 2a) and *M. fulvipes* (Fabricius, 1805) (Figure 3a) were model objects for the research.

### 2.1 Bee Behaviour and Plant Morphology

The behaviour of bees was registered using a Panasonic NV‐GS500 digital video camera featuring an additional photo shooting function (Panasonic Holdings Corporation, Kadoma, Osaka, Japan) and a GoPro HERO 10 Black high‐speed video camera (GoPro Inc., San Mateo, California, USA) with additional macro lenses. In total, more than 11 h of video recordings of bees' behaviour on flowers were captured and analysed. Frame‐by‐frame analysis of bee movements, which included about 35 thousand frames, was performed using the Pinnacle Studio 24 Ultimate programme (Corel Corporation, Ottawa, Canada). To analyse the external morphology of bees, including the oil‐collecting apparatus, as well as the allocation of collected oil and pollen on their bodies, 30 freshly collected females of *Macropis europaea* and 10 freshly collected females of *Macropis fulvipes* were examined. The difference in the number of specimens examined is due to the rarer occurrence of *M. fulvipes* . Fifty mature, fully oil‐filled CGTs from each of the three loosestrife species studied were also measured. Colour photographs of the external morphology of bees and flowers were taken using a Canon EOS 5D Mark‐II and a Canon EOS 5DS R cameras (Canon Inc., Tokyo, Japan) attached to a stereomicroscope Leica M205C with Leica LED5000 HDI illuminator (Leica Microsystems, Wetzlar, Germany) under Helicon Remote 3.9.10.w software. Photographs were stacked using Helicon Focus 8.2.0 Pro (Helicon Soft Ltd., Kharkiv, Ukraine) auto‐montage software and then edited using Adobe Photoshop CC, and Quick‐Photo Micro v2.3 (PROMICRA, s.r.o, Czech Republic) software was used for measurements. The ultrastructure of bee legs hairs and pollen grains was studied using scanning electron microscopy (SEM) with an SEM JEOL JCM‐6000 (JEOL Ltd., Tokyo, Japan) under Semaphore software (JEOL, Sollentuna, Sweden).

### 2.2 Histochemical Analysis of Flowers

*Lysimachia* flowers were collected at the initial flowering stage. For micromorphological studies, we used five flowers from each of the five plants belonging to three species (*N* = 75). For histochemical studies, we selected 10 flowers of *Lysimachia nummularia* , 10 flowers of *L. punctata*, and 7 flowers of *L. vulgaris* . Fresh flowers were fixed in Carnoy's fixative for 12 h to prepare microtome sections. The fixed plant material was washed from the fixative with three changes of 70% ethanol to remove any residual fixative. The flowers were then dehydrated in increasing concentrations of ethanol. The plant material was transferred to absolute ethanol, then to chloroform, and subsequently embedded in paraffin wax. Microtome sections (8 μm thick) of flower tissues (*N* = 30) were prepared from the paraffin blocks using a sledge microtome. To detect polysaccharides in the cells, the periodic acid‐Schiff (PAS) reaction was employed with the PAS staining system (Sigma‐Aldrich, cat. no. 395) (McManus 1948; Chawla et al. 2016). To establish the relationship between the qualitative composition, spatial distribution of polysaccharides, and the mechanical properties of trichomes that influence the availability of floral oil for bees, the qualitative composition of polysaccharides was determined using histochemical staining (PAS reaction) combined with selective enzymatic hydrolysis. Before staining, the microtome sections were deparaffinised. To remove pectins, the sections were treated with a 1.0 μM pectinase solution (Sigma‐Aldrich) in citrate buffer (pH 4.00) 25°C for 20 min in a humid chamber. The sections were then washed three times with distilled water and stained for polysaccharides. Stained sections without prior enzymatic hydrolysis served as controls.

The localisation of β‐galactosidase in the cells of glandular trichomes of *L. nummularia* was identified through histochemical staining with the substrate X‐Gal (5‐bromo‐4‐chloro‐3‐indolyl‐β‐D‐galactopyranoside) following a modified protocol of MacGregor et al. (1991). Flowers were fixed in 4.0% paraformaldehyde prepared in 0.1 M phosphate‐buffered saline (pH 7.3) 4°C for 2 h. After fixation, tissues were washed twice for 10 min each in the same buffer. The histochemical reaction was carried out 37°C for 4 h in 0.1 M acetate buffer (pH 4.8) containing 1.3 mM MgCl_2_, 3 mM potassium ferrocyanide (K_4_[Fe(CN)₆]), 3 mM potassium ferricyanide (K_3_[Fe(CN)_6_]), and 1 mg/mL X‐Gal dissolved in DMSO. Following staining, tissues were washed twice with acetate buffer and then rinsed in 40% ethanol. Enzyme localisation was visualised through the formation of a distinctive blue precipitate under light microscopy. As a negative control, fixed tissues were incubated in the reaction buffer without X‐Gal. The results of histochemical reactions were examined and photodocumented using a Nikon Eclipse E‐200 microscope equipped with a Nikon Coolpix L 830 camera (Nikon Corporation, Minato, Tokyo, Japan).

Fluorescence microscopy of the secretory structures of flowers was performed using an Olympus BX‐51 fluorescence microscope (Olympus Corporation, Tokyo, Japan) equipped with a Canon EOS 5D Mark II camera (Canon Inc., Tokyo, Japan).

The proportion of pectins in the polysaccharide complex was determined by comparing the intensity of the PAS reaction between the control and pectinase‐treated samples. To determine the proportion of hemicellulose, deparaffinised sections were treated with a 1.0 μM hemicellulase solution (Sigma‐Aldrich) in citrate buffer (pH 4.5) 40°C for 5 min. Subsequently, the sections were washed with distilled water and stained for polysaccharides. The localisation of polysaccharides in flower tissues was detected using standard protocols. Hemicellulose content in cell walls was determined for pectin, as previously described.

The intensity of histochemical reactions and the spatial distribution of polysaccharides in trichome structural elements were determined semi‐quantitatively using Image‐Pro Premier 9.0 software (Media Cybernetics, Rockville, MD, USA).

To study the distribution of polysaccharides and the effect of enzymatic hydrolysis, flowers were collected from different plants (*n* = 8), with each plant serving as an independent biological replicate. Polysaccharide staining intensity profiles were expressed in relative units (r.u.) based on pixel brightness values (0–255). Baseline polysaccharide intensity was measured across four different cell positions (neck cell, upper stalk cell, lower stalk cell, and basal cell) prior to enzymatic treatment. One‐Way Repeated Measures Analysis of Variance (RM ANOVA) with a post hoc Tukey test was used to identify significant differences among these four positions (*p* < 0.01).

To evaluate the structural heterogeneity of cells in CGTs (neck cell and stalk cell), enzymatic hydrolysis was used to examine the roles of pectins and hemicelluloses in the polysaccharide makeup of the cell walls. Intensity profiles were measured multiple times in both the neck cell and stalk cell, before and after treatment (separately for hemicellulase and pectinase). The data were analysed using Two‐Way Analysis of Variance (ANOVA) with Repeated Measures in XLSTAT (Addinsoft Inc., NY, USA, 2010). The analysis included two factors: cell position (neck versus stalk) and treatment (before versus after hydrolysis). The interaction term (cell position × treatment) was the main focus of the analysis, as it shows whether the hydrolysis effect depends on the cell position (indicating structural heterogeneity). Both factors (cell position and treatment) were treated as fixed effects. Differences were considered statistically significant at *p* < 0.01.

### 2.3 Mechanical Model of Oil Collection

For modelling the mechanical detachment of secretory heads, a brush composed of a bundle of elastic cylindrical nylon bristles (100–115 μm in diameter) with gradually tapered ends was used to imitate the collection of bee leg hairs. The perianth was carefully removed from freshly picked *Lysimachia nummularia* flowers, which were then fixed by the pedicel. The trichome heads were scraped from the surface of the staminal filaments using gentle, longitudinal motions from the base of the filament toward the anther. For sample transfer, the bristles were rinsed in microtubes containing 0.1 M acetate buffer (pH 4.8). The resulting suspension was pipetted onto microscope slides for subsequent microscopic analysis.

The principles of classical mechanics related to the destruction of materials (i.e., the conditions of equilibrium of the intensity of internal force or moments and external force or moments) were used to model the interaction between the bee's legs and the secretory structures of flowers, as well as to assess the strength of trichome cells. In the model, the main structural elements of the trichome were a multicellular secretory head containing oil and a two‐celled stalk. When constructing the model, we considered that during torsion, tangential stresses (τ) occur, which act in the plane of the cross‐section of the structure. During bending, normal stresses (σ) that act perpendicular to the cross‐section of the structure (measured as MPa, n/mm^2^ or Pa, n/m^2^) make the main contribution to the destruction of the structure. The level of maximum normal stresses during bending (*σ*), at which the structure collapses, is twice the level of maximum tangential stresses (*τ*) during torsion. Therefore, it is much easier to twist structures to the point of destruction than to bend them (Gere and Timoshenko 1997). We took this fact into account in our calculations of strength, the main idea of which is to choose the minimum permissible cross‐sectional area of the selected material structure and the given external stress using the so‐called condition of strength. In terms of tangential stresses (*τ*), the condition of strength is expressed as:

$$\tau_{\max\leq [}\tau_{]=}\;\sigma_{0.2}/3.0,$$

where *τ*_max_ is the maximum tangential stress that occurs in a given cross‐section of the structural material; *τ*_max_ = *М*_int_/*W*_ρ_, *М*_int_ is the maximum value of the internal torque that occurs in the cross section. *М*_int_ = *М*_ext_ = *R*·*F* ^sum^, where *М*_ext_ is the external torque applied transversely to the head of the trichome; and R is the maximum cross‐sectional radius of the trichome head. *F* ^sum^ is the total force applied transversely to the trichome head by the hairs on the bee's legs, *σ*_0.2_ is the yield strength of the material, the boundary between the areas of elasticity and plasticity of the material under tensile load, and 3.0 is the reserve factor.

Similarly, if there is a need for strength calculations based on normal bending stresses, the condition of strength is expressed as:

$$\sigma_{\max\leq [}\sigma_{]=}\;\sigma_{0.2}/1.5$$

where *σ*_max_ is the maximum normal stress that occurs in the given normal direction of the cross‐section of the structure during the external bending load, and [*σ*] is the permissible stress.

## 3 Results

### 3.1 Foraging Behaviour of *Macropis* Bees and Associated Micromorphological Features

**FIGURE 4:**
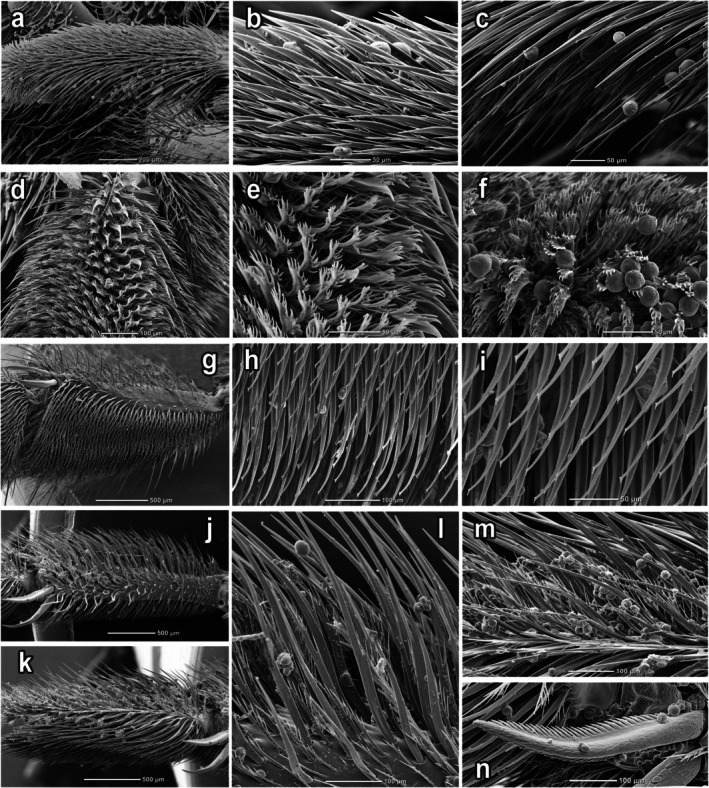
Pubescence of the legs of *Macropis europaea* Warncke, 1973 female: (a) Forebasitarsus in frontal view; (b) Hair on the outer side of the forebasitarsus; (c) Hair on the inner side of the forebasitarsus; (d–f) Hair on the outer side of the midbasitarsus; (g–i) Hair on the inner side of the metatibia; (j) Metatibia in frontal view; (k) Metabasitarsus in frontal view; (l) Hair on the outer side of the metatibia; (m) Hair on the outer side of the metabasitarsus; (n) Metatibial spur.

*Macropis* females possess long, comb‐ or brush‐like stiff hairs on the inner surface of their fore‐ and mid‐basitarsi and mid‐tibiae. We observed that they use these hairs to collect oil‐filled trichome heads from flowers and subsequently transfer them to the scopae on their hind legs (Figures 2, 3, 4). The stiff hairs are arranged in a manner that permits them to break off the oil‐filled trichome heads. In particular, the distance between the spine‐like hairs at their distal ends (25–35 μm; *n* = 50) is greater than the diameter of the trichome stalk (20–25 μm; *n* = 50) on which the spherical head (diameter 35–51 μm; *n* = 50) is situated, and only in the middle part of the hairs, the distance between them is approximately equal to the diameter of the trichome stalk. As a result of the sliding movements of the front and middle legs along the surface of the petals and stamens, the heads of the trichomes first break away by twisting, and then become lodged between the hairs (Figures 2c and 3d–f). Females tend to visit young, partly opened flowers with numerous oil‐containing trichome heads, as they haven't been visited by other bees yet.

**FIGURE 5:**
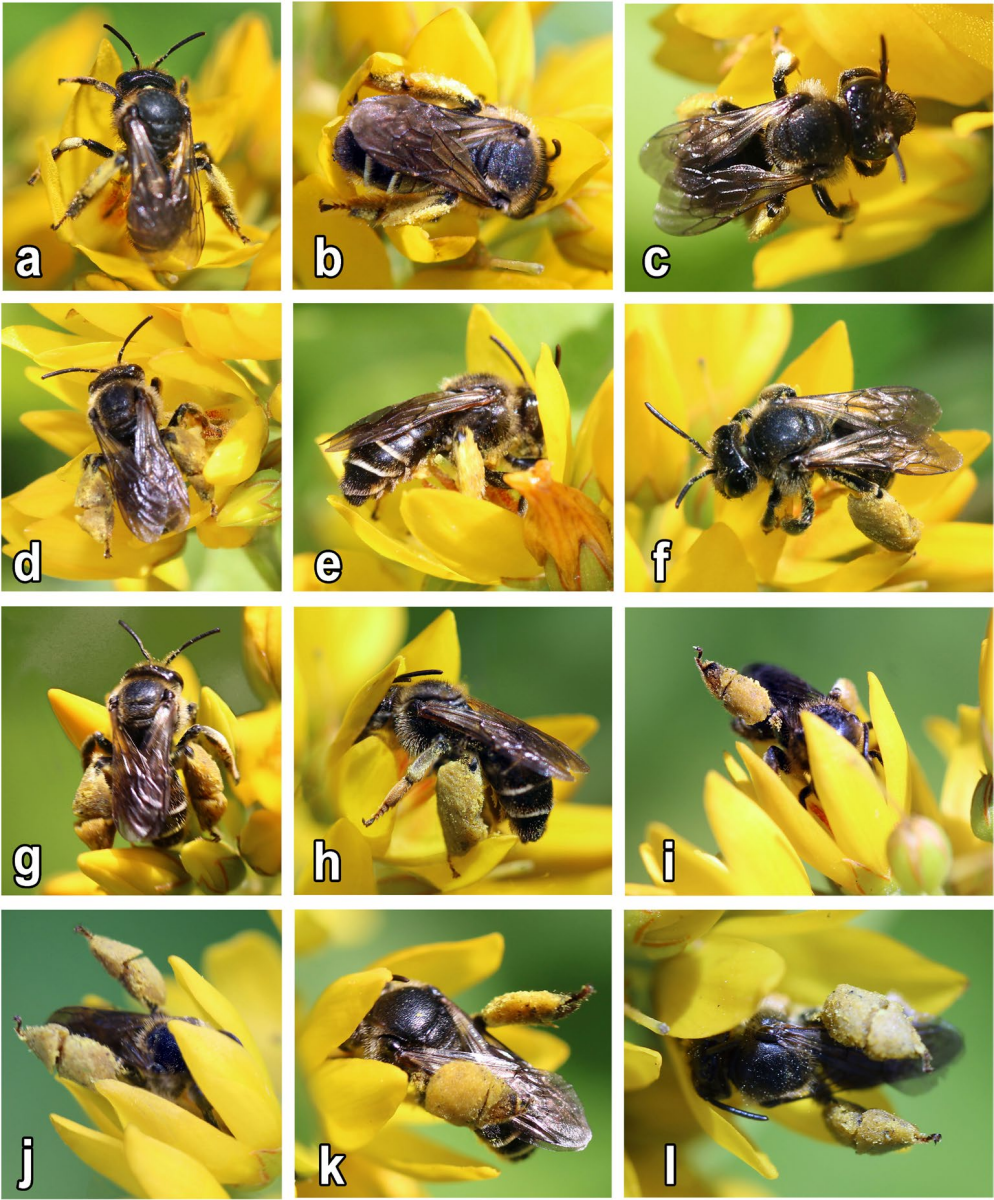
Behaviour of *Macropis* females on *Lysimachia* flowers: (a–h) Females use flower pistils and thick stamen columns as the axes to support their bodies while collecting pollen and floral oil; (i–l) Females with raised hind legs.

**FIGURE 6:**
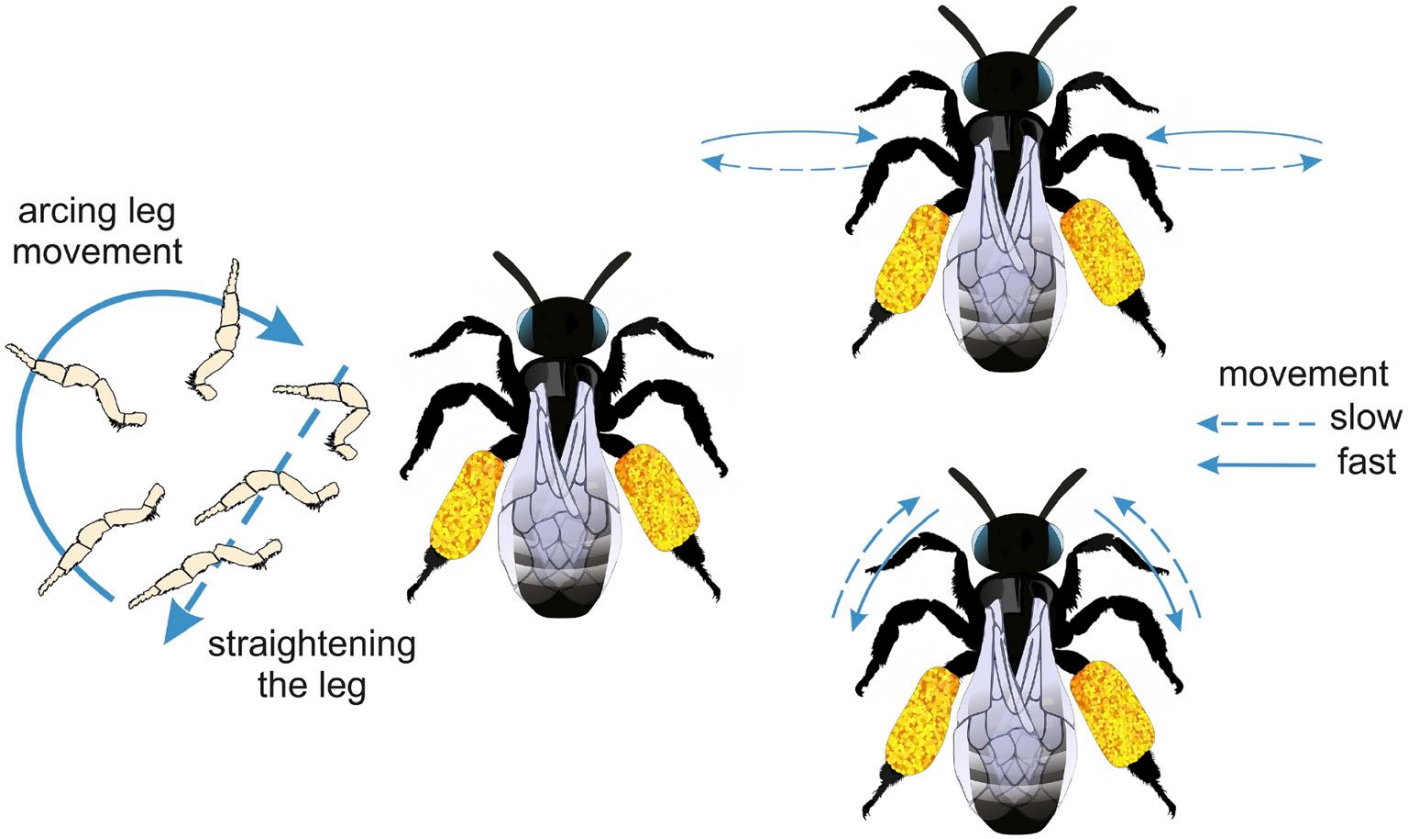
Movements of the legs of *Macropis* females during visits of *Lysimachia* flowers.

The process of collecting oil‐containing trichome heads from flowers is primarily ensured by the complex movements of the bee's middle and forelegs. Females of *Macropis europaea* and *M. fulvipes* , landing on a loosestrife flower, typically use the pistil of the flower and thick stamen columns as axes of support (Figure 5a–g). During the collection of oil, the bee, relying on such an axis, rotates around the pistil and stamens and, with semicircular or reciprocating movements of the front and middle legs (Figure 6), slides tightly along the inner surface of the flower petal, strewn with CGTs (Figure 1a–i), combing them out with the help of stiff hairs on its legs (Figure 2c). Females used similar motions to comb out the oil‐filled heads of CGTs located on the staminal columns.

After visiting a series of 3–5 flowers, the female carefully cleans off the accumulated oil‐filled trichome heads with gradual movements of its fore‐ and mid‐legs, transferring these heads and the pure oil spilled from the trichome heads to the scopae on its hind legs. In this process, the metabasitarsus is also involved. The collected oil‐filled trichome heads are stored among the hairs on the outer side of the forelegs and are subsequently transferred to the hairs on the inner surface of the middle legs. From there, the females move these trichome heads to the scopa located on the outer side of the hind legs. Similarly, the trichome heads are transferred from the inside of the front legs directly to the scopae. Additionally, with the help of the front legs, trichome heads with oil are transferred from the outer surface of the middle legs to the scopae on the hind legs. This transfer of oil happens both when the bee is on the flower and during its flight from one flower to another. The oil‐filled trichome heads and the oil spilled out from them are effectively retained in the scopae due to the special structure of its hairs.

**FIGURE 7:**
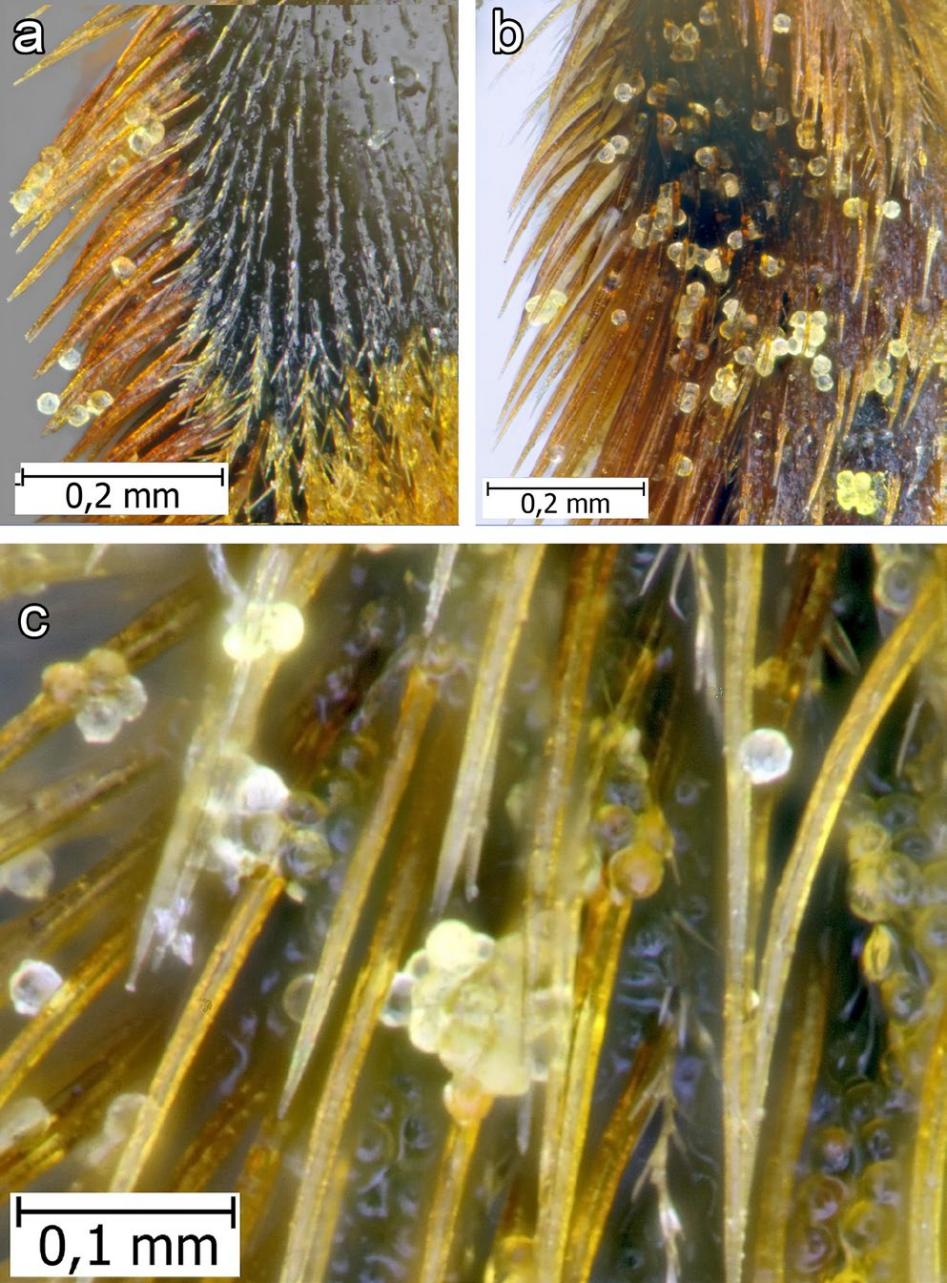
Broken‐off trichome heads filled with oil, found on the hairs of a female *Macropis fulvipes* (Fabricius, 1805) legs: (a) Midleg tibia; (b) Midbasitarsus; (c) Hind leg scopa.

The scopa consists of two types of hairs: longer, stiff, bristle‐like hairs and shorter, plumose, branched hairs that are intermixed with the longer ones (Figures 2h,i and 4l). In these hairs, females accumulate trichome heads with oil for further transfer into the nest. The micrographs clearly show the collected trichome heads on both the exterior of the hairs and deep within the pubescence of all legs, including scopae (Figure 7a–c). As a result, sufficiently large amounts of the oil fill the scopae, where it is kept from flowing out both due to its viscosity and the structure of the scopa hair (Figures 2h–i, 3h–i and 4l), which allows transferring the oil to the nest without loss. Foraging on floral oil without pollen is predominantly observed in the morning. Pure floral oil brought to the nest is used to waterproof line the walls of the new cells. The female collects pollen while sitting on a loosestrife flower and supported by the pistil and pollen‐bearing stamens, which causes the pollen to accumulate on the hairs on the ventral part of its body.

**FIGURE 8:**
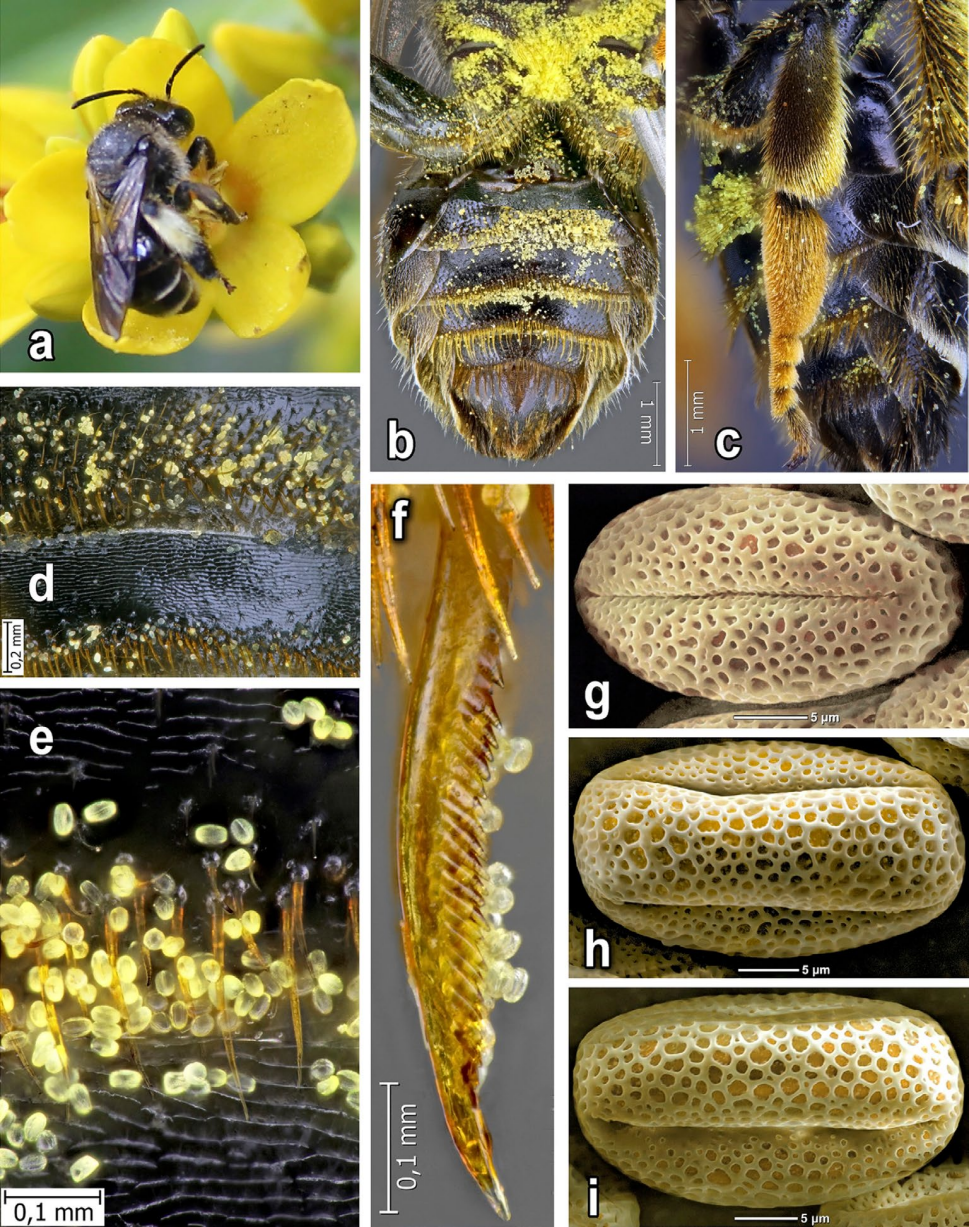
Features of behaviour and body structures for pollen collection in *Macropis* females: (a) Female of *M. europaea* that rests its ventral part of the body on the stamens of *Lysimachia nummularia* , resulting in the accumulation of a large amount of pollen in this part of the body; (b) Ventral part of female body with accumulated pollen; (c–e) Sternal bands with accumulated pollen of *Lysimachia*; (f) Metatibial spur of *M. europaea* female with *Lysimachia* pollen; (g–i) Dry pollen grains of *Lysimachia:* g— *L. vulgaris* L.; h— *L. punctata* L.; i— *L. nummularia* L.

The female collects pollen while sitting on the *Lysimachia* flower and supported by the pistil and pollen‐bearing stamens, which causes the pollen to accumulate on the hairs located on the ventral parts of its body (Figure 8a,b), ensuring cross‐pollination when visiting new flowers. Particularly, the bees *Macropis europaea* and *M. fulvipes* carry a significant amount of pollen on the hairs of the ventral part of the mesepisternum, coxae and femora of all legs (Figure 8b) and fixed in unusually widened, flat and branched hair at the top on the medbasitarsus outer side (Figure 4d–f), and also on eyelash‐like erect hairs that are located on the sterna marginal parts (Figure 8c–e).

Pollen is removed from the bee's body and transferred to the scopae on the hind legs using the front and middle legs, as well as with the inner spurs of the metatibiae (Figures 4n and 8f). Floral oil, along with pollen, is also transferred into the scopa, where it mixes with the pollen. The separate accumulation of pollen alone without adding oil to the scopa was not observed. As a result, quite large oil–pollen loads are formed on the scopae facilitated by the enlarged metabasitarsus at the *Macropis* females (Figures 2f,g,j and 3g).

However, it should be noted that such mutualism is not entirely complete, as *Lysimachia* plants do not secrete nectar, forcing *Macropis* bees to search for it on other plants. It is widely recognised that nectar serves as the primary energy source for bees. In the region we studied, bees primarily obtained nectar from *Cirsium arvense* (L.) Scop. flowers.

### 3.2 Histochemical Features of the Secretory Trichomes of *Lysimachia* Flowers

**FIGURE 9:**
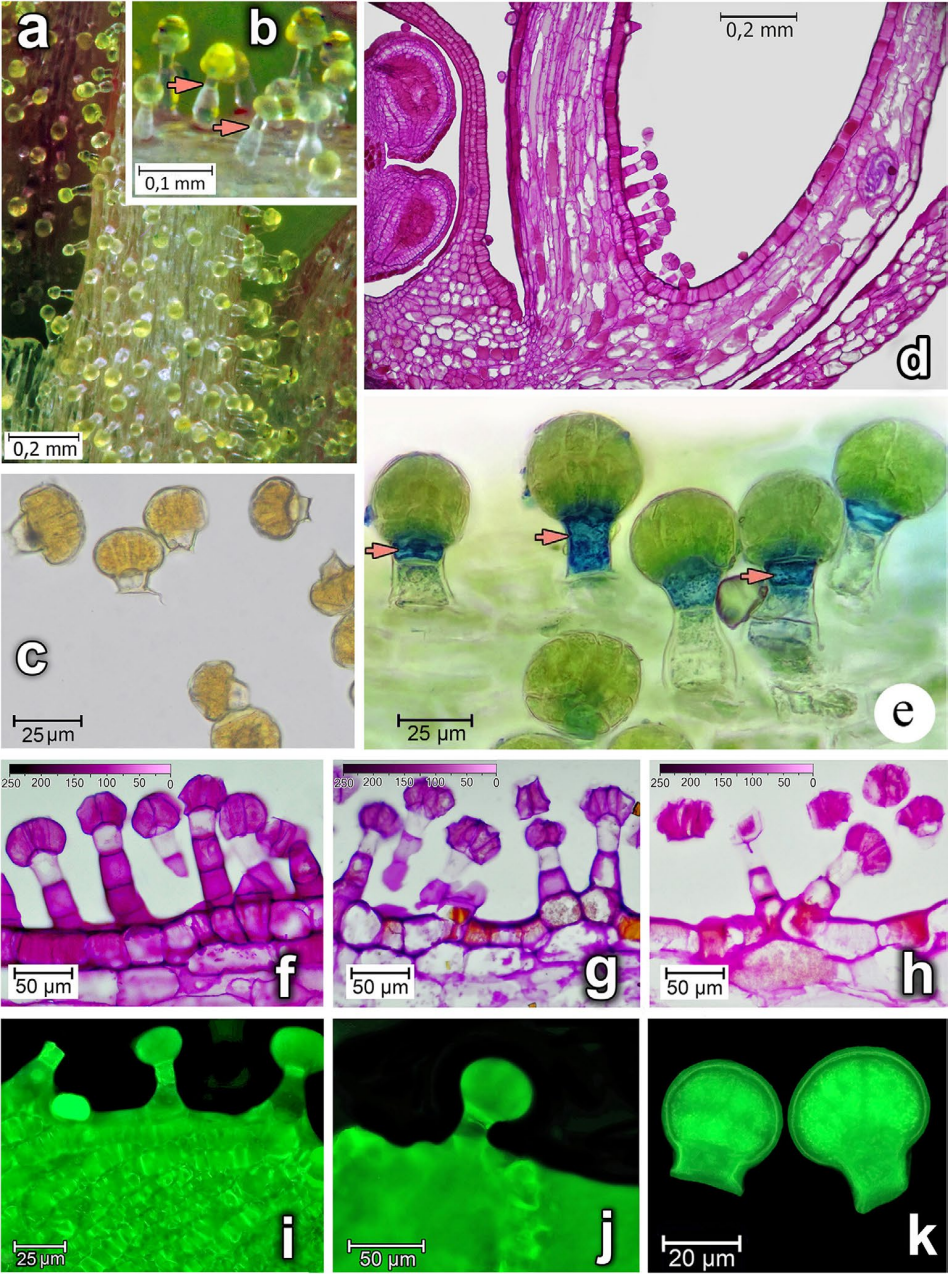
Trichomes of *Lysimachia* flowers, their structure and localisation of polysaccharides in them and the tissues of generative organs: (a) Part of flower stamen on which the trichomes with spherical oil‐filled heads are located; (b) Magnified trichomes with oil‐filled heads (the red arrows indicate the lower border of the upper stem cell, along which it detaches along with the trichome head.); (c) trichome heads broken off as a result of mechanical stress; (d) Polysaccharides in the tissues of the flower (longitudinal sections); e—Localisation of β‐galactosidase (BGAL) activity in the neck cells of capitate glandular trichomes on the surface of the stamen filament (detected using the X‐GAL substrate—blue staining). (f) Distribution of polysaccharides in cells of secretory trichomes; (g) Localisation of polysaccharides in the epidermis, middle and basal cells of trichome stems after enzymatic hydrolysis of pectin substances by pectinase; (h) Accumulation of polysaccharides in trichomes after enzymatic hydrolysis of tissues by xylanase and hemicellulase (periodic acid‐Schiff stain); (i, j) Autofluorescence of trichomes on the petal surface; (k) Autofluorescence of broken heads of *Lysimachia punctata* trichomes; a, b, k—*Lysimachia punctata;* c–h, − *L. nummularia;* i, j— *L. vulgaris* [f–h colour gradient scale reflects periodic acid‐Schiff staining intensity (r.u., pixel brightness 0–255)].

CGT of *Lysimachia* are multicellular secretory structures (Figure 9d,e). They consist of a stalk (2–4 cells) and a head (8 or 16 cells), which can synthesise and accumulate lipophilic substances and oils.

Given that the availability of floral oil is a vital condition for bee survival, we conducted a model test of the mechanical properties of the trichomes. During the test, the vast majority of trichomes demonstrated elastic properties: they bent under mechanical stress but fully recovered their original position, maintaining structural integrity. The destruction of the microstructure of individual trichomes occurred in two ways: either with the separation of the secretory head (Figure 9i) or with the separation of the head together with the neck cell of the stalk (Figure 9c,k).

Notably, no rupture of the heads or leakage of oil was observed during the experiment. Microscopic analysis of the petal surfaces and stamen filaments confirmed the presence of trichome stalks without heads, which were probably detached under natural conditions (Figure 9d,e,i). Both under natural conditions and in the experiment, head detachment occurred primarily at their junction with the neck cell or, less frequently, in the lower part of the same cell. Similar characteristics were observed in *Lysimachia punctata* (Figure 9a,b), where the neck cells are optically more transparent and are characterised by a reduced diameter (indicated by arrows) compared to the basal cell, which indirectly suggests a thin‐walled structure.

Microscopic examination of trichome cell wall thickness showed that they were thinnest in the neck cell specifically. These findings were supported by histochemical studies, which demonstrated differences in the structure and distribution of polysaccharides in the floral tissues and CGTs (Figure 9f–h).

**FIGURE 10:**
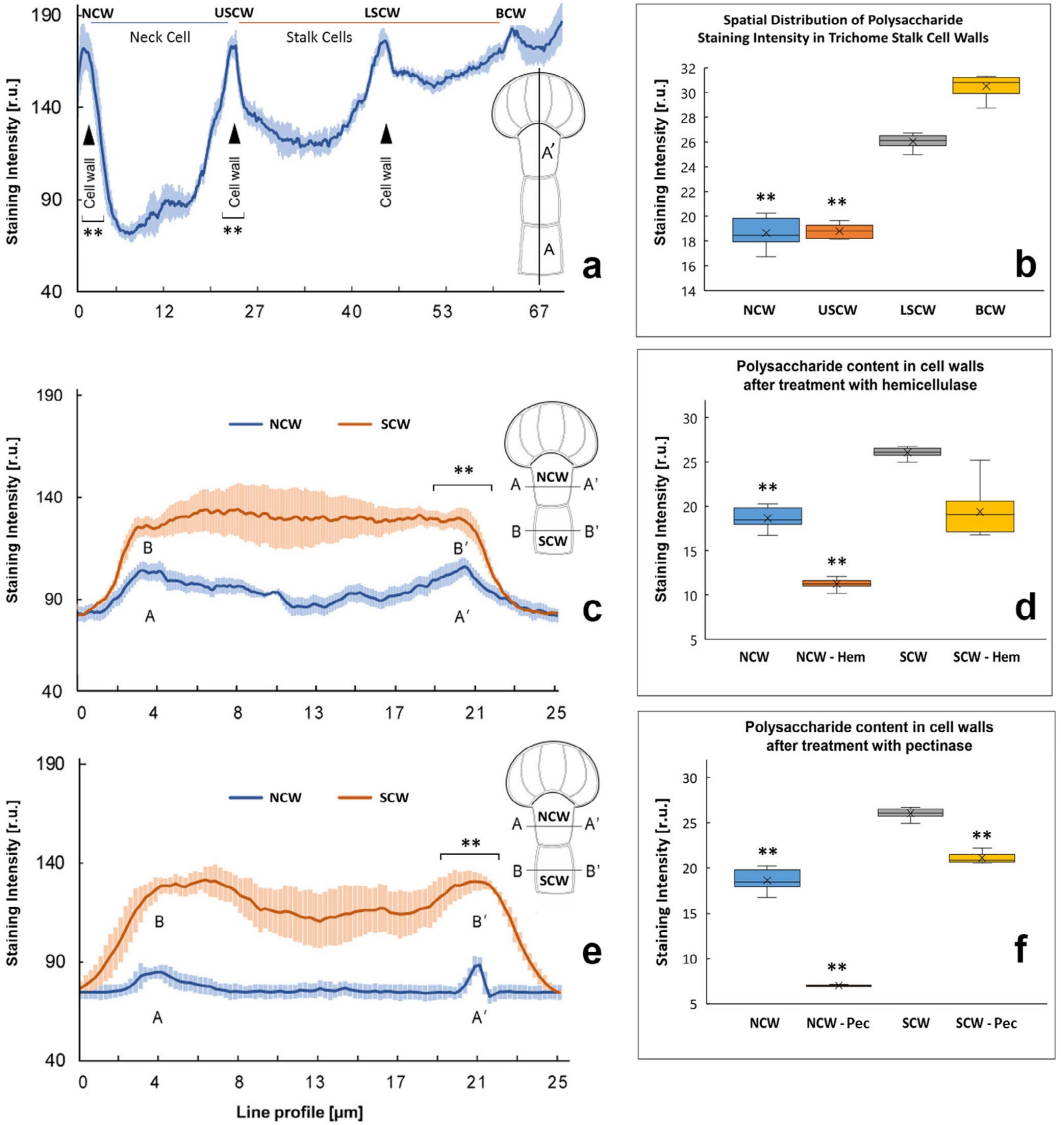
Distribution and quantitative analysis of polysaccharides in the cell walls of *Lysimachia nummularia* capitate trichome stalk cells: (a, b) Staining intensity in the cell walls of the neck (NCW), upper stalk (USCW), lower stalk (LSCW), and basal (BCW) cells; (c–f) Staining intensity in the cell walls of the neck (NCW) and stalk (SCW) cells before and after treatment with hemicellulase (Hem) (c, d) and pectinase (Pec) (e, f). Box plots represent the integrated staining intensity of the cell walls in relative units (r.u.). Statistical comparision: panels (a, b) were analysed using One‐Way Repeated Measures Analysis of Variance (RM ANOVA) with a post‐hoc Tukey test (HSD) panels (c–f) were analysed using Two‐Way RM ANOVA ** – indicates significant differences (*p* < 0.01) from: BCW (basal cell wall), used as control for panels (a, b); and SCW (stalk cell wall), used as control for panels (c–f).

Periodic acid‐Schiff (PAS) staining of polysaccharides highlighted notable differences in their content among the trichome cells (Figure 10a,b). It was determined that the reaction intensity in the cell walls of the neck cell (NCW) was 2.0–2.5 times lower than in the cell walls of the multicellular head, as well as the cells of the middle and basal parts of the stalk (Figure 10b). The most intense staining was observed in the basal stalk cell (BCW).

Regarding polysaccharides composition, it was found that after treatment with pectinase, the intensity of the PAS reaction decreased (Figure 10c–f), with the maximum reduction observed in the cell walls of the neck cell (NCW—Pec, Figure 10e,f). Treatment with hemicellulase also led to a reduction in trichome staining intensity (NCW—Hem), confirming the presence of pectin and hemicellulose in the cell walls. It was noted that the reduction in intensity following pectinase treatment was more pronounced than that after hemicellulase treatment (Figure 10c,d).

Based on the conducted Two‐Way Repeated Measures ANOVA, the proportion of pectins in the neck cell wall (NCW) statistically differed from that in the stalk cell wall (SCW), which is supported by a highly significant dependency of the enzymatic hydrolysis effect on the cell position (*p* < 0.001). In contrast, hemicellulase showed a similar effect across both cell types (*p* = 0.509). Pectinase treatment caused a significant reduction in the histochemical reaction signal in the neck cells (approx. 63% reduction) compared to the stalk cells (approx. 19% reduction). This key finding supports the hypothesis of structural heterogeneity and localised susceptibility to degradation of the neck cells, particularly under conditions involving the activation of enzyme systems associated with cell wall remodelling.

Given the observed difference in CGT's ability to withstand mechanical stress, we analysed the level of β‐galactosidase activity, which significantly influences the composition and spatial structure of heteropolysaccharides, their degree of cross‐linking, and consequently, the mechanical properties of the cell walls. To determine the intracellular localisation and activity of β‐galactosidase, freshly collected flowers were used: after appropriate fixation, trichomes were stained with X‐Gal (5‐bromo‐4‐chloro‐3‐indolyl‐β‐D‐galactopyranoside).

Based on the presence or absence of the blue reaction product in the cells, it was determined that β‐galactosidase activity is not detected in all CGT. The spatial distribution of CGT on the stamen filament showed a clear gradient, with the proportion of trichomes containing active enzyme reaching 75% in the basal part of the stamen filament, but decreasing to 15% nearer the anthers, along with a corresponding reduction in the total number of trichomes.

An important feature was that high galactosidase activity was detected exclusively in the neck cells. The product of the enzymatic reaction was mainly localised at the junction between the stalk cell and the head (Figure 9). Our results provide a comprehensive picture of the spatial distribution and transformation of the polysaccharide complex within the multicellular secretory structure during its maturation.

### 3.3 Mechanics of Breaking Off Heads of Trichomes by Stiff Hairs on the Bee's Legs

In the context of the structural elements of the flower, strength refers to the ability of the CGT to withstand the external load imposed by the pubescence on the bee's legs without breaking. Along with strength, this bioconstruction must possess sufficient rigidity to endure a predetermined deformation.

**FIGURE 11:**
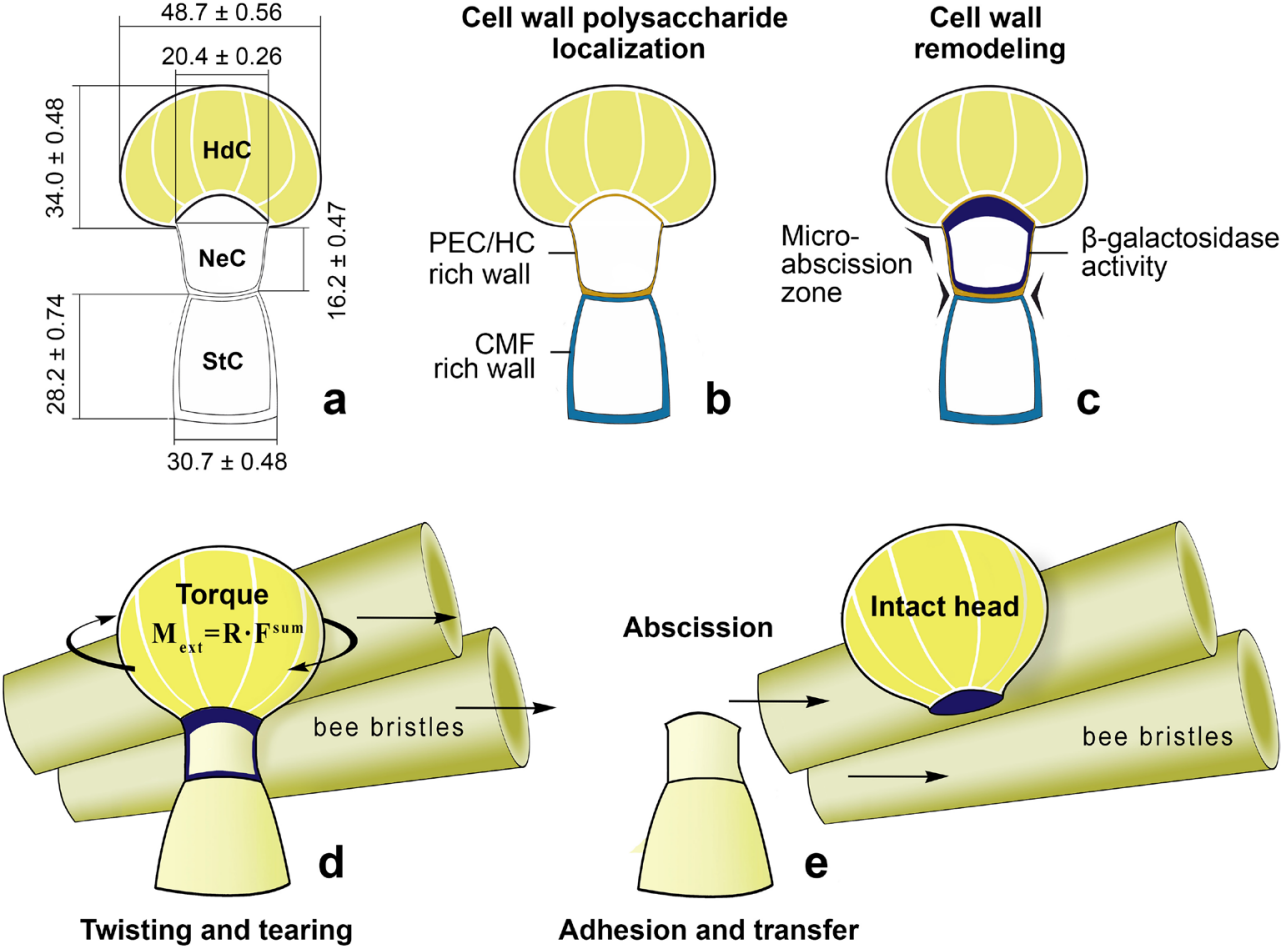
Schematic representation of the *Lysimachia* capitate trichome structure and the biomechanical model of head microabscission by *Macropis* bees: (a) capitate trichome morphometry (longitudinal section): Stalk cell (StC), neck cell (NeC) and multicellular head (HdC); (b) polysaccharide distribution in cell walls: CMF‐rich—stalk cell (StC) walls rich in cellulose microfibrils (CMF), ensuring their high tensile strength, PEC‐HC‐rich—neck cell (NeC) walls rich in pectins (PEC) and hemicellulose (HC), forming a matrix with low shear stiffness (high PEC‐HC/CMF ratio); (c) scheme of spatial localisation of β‐galactosidase (BGAL) activity, detected using the X‐GAL substrate (signal restricted to NeC); (d) trichome rupture mechanics at the moment *Macropis* bristles apply a torsional load (Torque) to the head; (e) resource collection: Adhesion and transfer of the intact secretory head (HdC) by the bristles on the bee's leg.

According to the results in Section 2.3, the maximum normal and shear stresses in the most critical sections of any structural element should always be below the permissible stresses. Based on the initial structural parameters of trichomes, the safety factors for their structural elements concerning normal and shear stresses vary by a factor of 2 (1.5 and 3.0). Consequently, the permissible stress levels [*σ*] and [*τ*] will also differ by a factor of 2. Since normal stresses dominate under bending conditions, it can be argued that twisting a trichome head is considerably easier than breaking it by bending (Figure 11).

The maximum tangential stress of structural material is defined as *τ*_max_ = М_int_/W_ρ_, here М_int_ is the maximum value of the internal torque that is the most critical.

The external torque applied transversely to the head of the trichome is equal to *М*_int_ = *М*_ext_ = *R*•*F* ^sum^, where *R* is the mean radius of the cross‐section of the trichome head (based on measurements, *R* = 21.5 μm). *F* ^sum^ is the total force exerted in the transverse direction on the head of the trichome by the bristles on the front or middle legs of bees. Each bristle creates a separate load in relation to the cell breaking; when several bristles press on the head of the trichome, the *F* ^sum^ indicator increases accordingly, as it represents the sum of the forces acting on each bristle.

To balance the external torque applied to the head of the trichome, the upper cell of the trichome stalk (averaging 17.0 μm in length, according to the measurements obtained) possesses an internal torque M_int_, which is equal to the external torque *M*_ext_: *M*_int_ = *M*_ext_.

The internal torque *M*_int_ remains constant along the entire length of the trichome stalk. To determine the level of maximum tangential stresses in the minimum cross‐section of the trichome stalk *τ*_max_ at a distance of 17.0 μm, we calculate the ratio of *M*_int_ to the polar moment of resistance *W*_ρ_. When the cross‐section of a trichome stalk cell exhibits the same mechanical properties, then:

$$W_{\rho }=\pi \;r^{3}/2,$$

where *r* is the radius of the minimum cross‐section of the trichome stalk (according to the obtained data, *r* = 7.75 μm). It is noted that the cell of the trichome stalk has a tubular cross‐section and that the material inside offers practically no resistance to rotation, thus:

$$W_{\rho }=\pi \;\left({r_{1}}^{3}–{r_{2}}^{3}\right)/2,$$

where *r*_1_ and *r*_2_ represent the radii of the tubular section, respectively.

The analysis of the relevant formulas indicates that since the radius (*r*) of the intermediate cell of the stalk (neck cell) is 2.8 times smaller than the head radius (*R*), and the significant torque value is divided by the smaller polar moment of resistance, the maximum tangential stresses concentrate in this zone of the trichome stalk cell, exceeding the allowable limits. If the condition for torsional strength is not satisfied, and the breaking mechanism of the trichome stalk will rupture at its thinnest and weakest point of its polysaccharide frame, specifically at the neck cell. Considering the average value [*σ*] of the bristles on bee legs, it is also possible to determine the number of bristles needed to detach the heads containing oil, based on the geometric shape and size of the trichomes.

We have proposed a theoretical model for measuring the average value [*σ*] of bristles on bee legs, which can be very easily tested experimentally if suitable equipment is available that allows the study to be conducted at extremely small values of [*σ*].

From the perspective of the efficiency of the bee's movements, collecting oil by tearing off the heads of the trichomes is the most economical method, as it prevents oil from spreading onto the surfaces of the flower. This is achievable only under specific conditions related to the consistency in the structure of the cell walls of the trichomes and the movement of the legs covered with specialised hairs.

## 4 Discussion

Our study introduces, for the first time, a biomechanical model of micro‐abscission of trichome heads in *Lysimachia nummularia* , *L. punctata* , and *L. vulgaris* by *Macropis* bees. It demonstrates that the easier detachment of ‘mature’ oil‐containing trichome heads results from sequential physiological processes within the plant rather than random mechanical rupture of the apical cell walls of the trichome stalk.

The mechanics of floral oil collection by bees largely depend on the micromorphology and strength of the structural elements of secretory trichomes. In this regard, different descriptions of this process exist. For example, when *Tapinotaspis* sp. bees collect floral oil from the trichome elaiophores of *Angelonia* (Scrophulariaceae) flowers, they use absorptive pads with long, plumose, pinnate hairs on the female's midlegs that suck up and store the secretion prior to its deposition in the scopae (Machado et al. 2002). A brush of fine feathery hairs is remarkably developed. Epithelial elaiophores may rupture on the surface when the bees of the genera *Centris, Epicharis* and *Monoeca* interact with them (Martins et al. 2015; Possobom and Machado 2018). The oil chamber of the secretory head in *Nierembergia* flowers bursts spontaneously due to the rupture of the cuticle when the flowers open and their hairs are exposed to sunlight. A slight touch also causes the flower hairs to burst (Cocucci 1991). Observations of glands after bee visitation clearly show that during the collection process, female *Centris* rupture the cuticles while scraping the surface (Neff and Simpson 1981). Regarding bees of the genus *Macropis*, Scheffler and Dotterl noted that females collect oil by touching the stamen columns and the bases of the petals, where most of the oil glands are situated, with the pads of their middle and fore legs. Therefore, Scheffler and Dotterl (2011) believe that the floral oil is absorbed through capillary action. However, based on our results, the collection of oils from *Lysimachia* flowers by *Macropis* bees seems to be a more intricate mechanical process. Furthermore, we challenge the prevailing viewpoint that bees collect pure oil directly from *Lysimachia* flowers.

Our observations demonstrate for the first time that bees detach and collect the oil‐filled trichome heads rather than pure oil, representing a more efficient foraging strategy. The bees use specialised stiff hairs on their fore and middle legs to scrape oil‐rich trichome heads from the surfaces of petals and stamens. The structure and arrangement of these specialised hairs in *Macropis* have been partially described previously (Roberts and Vallespir 1978; Cane et al. 1983; Michez and Patiny 2005). These structures appear somewhat simpler compared to the corresponding features found in species of the genus *Centris* Fabricius, 1804, and some Ctenoplectrini (Roberts and Vallespir 1978). Roberts and Vallespir (1978) reported that the primary mass of flower oil in these bees is gathered on the short, slender, and flexible hairs with numerous branches, while the long, stiff, unbranched hairs serve as an additional axis for holding the pollen mixed with oil. In addition, such bees have hairs that are capable of scraping up oil (Roberts and Vallespir 1978; Buchmann and Buchmann 1981; Cocucci 1991; Vogel and Machado 1991; Vogel and Cocucci 1995; Steiner and Whitehead 2002).

Our study reveals a complex array of traits in the structure of multicellular oil‐secreting trichomes of *Lysimachia* species that facilitate oil collection by bees. This point becomes more convincing when compared to the trichomes of *Lysimachia arvensis* (L.) U. Manns and Anderb, which have a distinctly different structure. They do not accumulate oil, possess a three‐celled structure with a relatively small unicellular head, and are located at the edge of the petals. Some believe that these trichomes attract pollinating insects (Rhizopoulou et al. 2015). Accordingly, such structures should be mechanically stronger, aligning with their attracting function and the fact that they are frequented by generalist bees that are not adapted to oil collection (Schäffler et al. 2012).

The process of oil collection by bees of the genus *Macropis* from trichome‐type elaiophores in *Lysimachia* appears to demonstrate a highly specialised co‐adaptative process, where the complex behavioural responses of the pollinator are notably coordinated with the structure and physiological processes of the flowers.

To understand the mechanism behind floral oil collection by bees, it is important to examine the specific structure of trichome cell walls, which largely determines their strength. Microscopic and histochemical studies have revealed key structural features of CGT stalk cells. These mainly relate to variations in cell wall thickness within the stalk cells. In the basal and lower stalk cells, as well as in the cells of the multicellular head, they were significantly (1.5–2 times) thicker than in the intermediate (neck) cell. The polysaccharide composition of the cell walls also differed considerably. Neck cells showed higher pectin and hemicellulose content with a relatively low cellulose content (Figure 10). A reduction in the proportion of cellulose components, which are mainly responsible for cell wall rigidity, weakens cells. According to Scheller and Ulvskov (2010), it is the cellulose microfibrils that determine the primary tensile strength of the cell wall. This is because the microfibrils' integrity under axial tension is preserved by strong covalent bonds between the glucan chains, which have a very high tensile modulus (E). Resistance to shear or torsion is offered by much weaker hydrogen bonds and non‐covalent interactions between adjacent chains and the polymer matrix. Furthermore, the calculated shear modulus (G) in the azimuthal direction is almost two orders of magnitude lower than the tensile modulus (E) (Zhao et al. 2013).

A high proportion of hemicelluloses, particularly xyloglucans, xylans, and pectins, ensures the elasticity and structural integrity of the cell wall (Scheller and Ulvskov 2010; Cheng et al. 2020). According to Cruz‐Valderrama et al. (2021), pectins are involved in maintaining cell shape and intercellular communication. A reduction in their content decreases the cohesion of the biopolymer scaffold, weakens the adhesion forces between cells, and facilitates their separation. However, these components cannot provide cell walls with mechanical strength comparable to that of cellulose microfibrils.

This enzyme mainly degrades pectin galactans and hemicelluloses (Brummell 2006; O'Donoghue et al. 2009). Since pectins and hemicelluloses dominate in the apical cell walls of the CGT, the polysaccharide complex they form can be significantly modified and broken down by BGal. The reduced connectivity of the polysaccharide matrix and microfibrils caused by side chains decreases the mechanical strength of the cell walls in CGTs, making them even more susceptible to shear stress (with a low calculated shear modulus G), especially in the area of enzyme activity. Since the laws of materials science and mechanics also apply to polysaccharides and other biopolymers, the mechanical properties of trichomes can be further analysed using established physical principles (Kaletnik et al. 2011). Therefore, the torque generated by the tension of the bee bristles overcomes the residual shear stiffness of the weakened cellulose framework of the intermediate cell and leads to the inevitable detachment of the head along the azimuthal plane. This effect is evidenced by the extensive accumulation of trichome heads on the hind legs of *Macropis* (Figures 3h,i and 7e). Beneath the bristles, rounded multicellular structures measuring 40–50 μm are distinctly visible, featuring a darkened, rounded zone in the centre, which, in both size and position, aligns with the region of trichome microabscission.

Therefore, the separation of the multicellular head in *Lysimachia nummularia* represents a fundamentally different biological process from simple mechanical rupture or decapitation of trichomes with the release of toxic metabolites for protection against phytophages (Glas et al. 2012). The mechanism described above is particularly important in the interaction of *Lysimachia* plants with oil‐collecting bees, as it leaves the flower's covering undamaged and reduces the risk of infection by dangerous pathogens. An example of plant infection by a dangerous pathogen ( *Erwinia amylovora* ) following ethylene‐induced abscission of multicellular trichomes was described for *Malus domestica* (Millett et al. 2025). Plants become infected through wounds where they detach from the leaf blade. Trichome heads in *Lysimachia* flowers are separated through a prepared area, virtually preventing damage to the flower's integumentary tissues. Functionally, the detachment of the oil‐filled multicellular trichome head at its junction with the apical cell of the stalk can be characterised as microabscission, as the detachment zone is defined by enzymatic modification of the cell wall. This mechanism notably facilitates oil collection by pollinators and serves as a beneficial adaptation for the plant. In the context of mutualism, the phenomenon of trichome microabscission likely enhances the ecological flexibility of *Lysimachia* plants by supporting their specialised interactions with oil‐collecting bees.

## 5 Conclusion

*Lysimachia* plants produce capitate glandular trichomes on the surfaces of petals and stamen columns. The shape, size, and structural features of these trichomes enable bees to collect their secretory heads, which contain oil and other highly active compounds. The cell walls of the upper cells of the trichome stalk are thinner than those in other parts, and the proportion of pectins in their polysaccharide matrix is increased. This part of the trichome's structure exhibits the least strength. When exposed to external mechanical forces, this cell fractures along the line of the lower or upper anticlinal wall. The bee presses and pulls its front and middle legs against the surfaces of the petals and the stamen columns, which are covered with numerous trichomes. In fully matured trichomes, where the cell walls of the upper stalk cells are loosened, the head easily detaches under the slightest force. In relation to other trichomes, torque is generated due to the adhesion of the sticky surfaces of the heads to the elastic hairs on the legs of the bee during gliding movements. Given the nearly threefold difference in the radii of the head and the upper cell of the stalk, significant tangential stresses arise in the latter, exceeding permissible limits. Consequently, the condition for torsional strength is not met, leading to the cell of the trichome stalk breaking at the thinnest point. The twisted and separated heads cling to the hairs on the bee's legs. With repeated movements, the broken heads of trichomes are pushed deeper into the tarsus surface. After collecting sufficient plant material, the bee transfers it to the scopae on its hind legs.

Furthermore, we challenge the prevailing viewpoint that *Macropis* bees collect pure oil directly from *Lysimachia* flowers. Our observations reveal for the first time that female *Macropis* bees do not just collect pure oil, but instead gather oil‐filled trichome heads from *Lysimachia*, which indicates a more efficient foraging strategy. Therefore, the widespread belief that bees acquire pure oil solely through capillary absorption via hair pads on the tarsi of their fore and middle legs—wiping it directly from trichomes in *Lysimachia* flowers—is incorrect.

The differences in the mechanical strength of the cell walls of individual structural elements of the capitate trichomes of *Lysimachia* contribute to the effective collection of floral oil by *Macropis* bees.

Thus, the co‐adaptation of pollinator insects and plants ensures the collection of flower oil in sufficient quantities for nest building and provisioning of bee larvae, while plants benefit from reliable pollination. This co‐adaptation is reflected in the consistency of the structure of trichome joints and the regulation of physiological processes in the flowers of the host plant on one side, alongside the specific behaviour of bees that enables the effective use of the stiff hairs on the tibiae and tarsi of the front and middle legs on the other. Consequently, the narrow adaptation of *Macropis* bees to *Lysimachia* plants reliably promotes their cross‐pollination, thereby increasing the genetic heterogeneity of the plant population. This is a clear example of co‐adaptation within the narrow plant–pollinator system.

## Author Contributions

**Vladimir G. Radchenko:** conceptualization (equal), data curation (equal), formal analysis (equal), investigation (equal), methodology (equal), project administration (lead), resources (equal), supervision (lead), validation (equal), visualization (equal), writing – original draft (equal), writing – review and editing (equal). **Mykola G. Chausov:** data curation (equal), formal analysis (equal), investigation (equal), methodology (equal), resources (equal), visualization (equal), writing – original draft (equal). **Artur F. Likhanov:** conceptualization (equal), data curation (equal), formal analysis (equal), investigation (equal), methodology (equal), resources (equal), validation (equal), visualization (equal), writing – original draft (equal), writing – review and editing (equal). **Hanna Yu. Honchar:** data curation (supporting), formal analysis (supporting), investigation (equal), resources (equal), visualization (equal), writing – original draft (supporting). **Denis Michez:** conceptualization (equal), data curation (equal), formal analysis (equal), investigation (equal), validation (equal), visualization (equal), writing – original draft (equal), writing – review and editing (equal).

## Funding

The authors have nothing to report.

## Conflicts of Interest

The authors declare no conflicts of interest.

## Supporting information


**Data S1:** Supporting information.

**Data S2:** Supporting information.

## Data Availability

Data are available as additional supporting information, accessible online in Data S1 and S2.

## References

[ece372544-bib-0001] Alves‐dos‐Santos, I. , S. Naxara , and E. F. L. R. A. Patrício . 2006. “Notes on the Morphology of Tetrapedia Diversipes Klug 1810 (Tetrapediini, Apidae), an Oil‐Collecting Bee.” Brazilian Journal of Morphological Sciences 23, no. 3–4: 425–430.

[ece372544-bib-0002] Asar, Y. , S. Y. W. Ho , and H. Sauquet . 2022. “Early Diversifications of Angiosperms and Their Insect Pollinators: Were They Unlinked?” Trends in Plant Science 27, no. 9: 858–869. 10.1016/j.tplants.2022.04.004. 35568622

[ece372544-bib-0003] Ascher, J. S. , and J. Pickering . 2024. “Discover Life Bee Species Guide and World Checklist (Hymenoptera: Apoidea: Anthophila).” Accessed 10 December 2024. https://www.discoverlife.org/mp/20q?guide=Apoidea_speciesandflags=HAS.

[ece372544-bib-0004] Bartomeus, I. , J. S. Ascher , D. Wagner , et al. 2011. “Climate‐Associated Phenological Advances in Bee Pollinators and Bee‐Pollinated Plants.” Proceedings of the National Academy of Sciences of the United States of America 108, no. 51: 20645–20649. 10.1073/pnas.111555910. 22143794 PMC3251156

[ece372544-bib-0005] Bassin, L. , N. Alvarez , L. Pellissier , and Y. Triponez . 2011. “Ecological Niche Overlap in Sister Species: How Do Oil‐Collecting Bees *Macropis europaea* and *Macropis fulvipes* (Hymenoptera: Melittidae) Avoid Hybridization and Competition?” Apidologie 42, no. 5: 579–595.

[ece372544-bib-0105] Bergau, N. , S. Bennewitz , F. Syrowatka , G. Hause , and A. Tissier . 2015. “The Development of Type VI Glandular Trichomes in the Cultivated Tomato *Solanum lycopersicum* and a Related Wild Species *S. habrochaites* .” BMC Plant Biology 15: 289. 10.1186/s12870-015-0678-z. 26654876 PMC4676884

[ece372544-bib-0006] Bogusch, P. , E. Bláhová , and J. Horák . 2020. “Pollen Specialists Are More Endangered Than Non‐Specialised Bees Even Though They Collect Pollen on Flowers of Non‐Endangered Plants.” Arthropod–Plant Interactions 14, no. 6: 759–769. 10.1007/s11829-020-09789-y.

[ece372544-bib-0106] Bossert, S. , E. A. Murray , E. A. B. Almeida , et al. 2019. “Combining Transcriptomes and Ultraconserved Elements to Illuminate the Phylogeny of Apidae.” Molecular Phylogenetics and Evolution 130: 121–131. 10.1016/j.ympev.2018.10.012. 30326287

[ece372544-bib-0007] Boucher, F. C. , N. E. Zimmermann , and E. Conti . 2016. “Allopatric Speciation With Little Niche Divergence Is Common Among Alpine Primulaceae.” Journal of Biogeography 43, no. 3: 591–602. 10.1111/jbi.12652.

[ece372544-bib-0008] Bronstein, J. L. , R. Alarcón , and M. Geber . 2006. “The Evolution of Plant–Insect Mutualisms.” New Phytologist 172, no. 3: 412–428. 10.1111/j.1469-8137.2006.01864.x. 17083673

[ece372544-bib-0009] Brummell, D. A. 2006. “Primary Cell Wall Metabolism During Fruit Ripening.” New Zealand Journal of Forestry Science 36, no. 1: 99–111.

[ece372544-bib-0010] Buchmann, S. L. , and M. D. Buchmann . 1981. “Anthecology of *Mouriri myrtilloides* (Melastomataceae: Memecyleae), an Oil Flower in Panama.” Biotropica 13, no. 2: 7. 10.2307/2388066.

[ece372544-bib-0011] Buchmann, S. L. 1987. “The Ecology of Oil Flowers and Their Bees.” Annual Review of Ecology and Systematics 18, no. 1: 343–369. 10.1146/annurev.es.18.110187.002015.

[ece372544-bib-0012] Burger, H. , S. Dötterl , C. M. Häberlein , S. Schulz , and M. Ayasse . 2012. “An Arthropod Deterrent Attracts Specialised Bees to Their Host Plants.” Oecologia 168, no. 3: 727–736. 10.1007/s00442-011-2136-4. 21964494

[ece372544-bib-0014] Cane, J. H. , G. C. Eickwort , F. R. Wesley , and J. Spielholz . 1983. “Foraging, Grooming and Mate‐Seeking Behaviors of *Macropis nuda* (Hymenoptera, Melittidae) and Use of *Lysimachia ciliata* (Primulaceae) Oils in Larval Provisions and Cell Linings.” American Midland Naturalist 110, no. 2: 257–264. 10.2307/2425267.

[ece372544-bib-0107] Cappellari, S. C. , H. Schaefer , and C. C. Davis . 2013. “Evolution: Pollen or Pollinators – Which Came First?” Current Biology 23, no. 8: R316–R318. 10.1016/j.cub.2013.02.049. 23618666

[ece372544-bib-0015] Cardinal, S. , and B. N. Danforth . 2013. “Bees Diversified in the Age of Eudicots.” Proceedings of the Royal Society B: Biological Sciences 280, no. 1755: 20122686. 10.1098/rspb.2012.2686. PMC3574388 23363629

[ece372544-bib-0016] Carneiro, L. T. , C. B. D. S. André , A. Takahasi , and I. Alves‐dos‐Santos . 2019. “Interactions Between Oil‐Collecting Bees and Krameria Grandiflora (Krameriaceae) With Emphasis on the Role of Specialized Floral Traits in the Mutual Fit.” Arthropod‐Plant Interactions 13: 213–226. 10.1007/s11829-019-09689-w.

[ece372544-bib-0017] Carneiro, L. T. , A. A. Cocucci , A. N. Sérsic , I. C. Machado , and I. Alves‐Dos‐Santos . 2024. “Pollinator‐Mediated Selection on Krameria Oil Flowers: A Flower–Pollinator Fit Adaptation to an Atypical Oil‐Collecting Behaviour?” Annals of Botany 134: 603–614. 38916514 10.1093/aob/mcae102 PMC11523623

[ece372544-bib-0018] Carneiro, L. T. , and I. C. Machado . 2023. “Oil Flowers and Related Oil‐Collecting Bees: A 50‐Year Timeline of Knowledge and Future Directions.” Arthropod‐Plant Interactions 17: 543–562. 10.1007/s11829-023-10000-1.

[ece372544-bib-0019] Celary, W. 2004. “A Comparative Study on the Biology of *Macropis fulvipes* (Fabricius, 1804) and *Macropis Europaea* Warncke, 1973 (Hymenoptera: Apoidea: Melittidae).” Folia Biologica‐Krakow 52, no. 1–2: 81–85. 15521653

[ece372544-bib-0020] Chawla, M. , V. Verma , M. Kapoor , and S. Kapoor . 2016. “A Novel Application of Periodic Acid‐Schiff (PAS) Staining and Fluorescence Imaging for Analysing Tapetum and Microspore Development.” Histochemistry and Cell Biology 147, no. 1: 103–110. 10.1007/s00418-016-1481-0. 27565968

[ece372544-bib-0021] Cheng, G. , L. Wang , S. He , J. Liu , and H. Huang . 2020. “Involvement of Pectin and Hemicellulose Depolymerization in Cut Gerbera Flower Stem Bending During Vase Life.” Postharvest Biology and Technology 167: 111231. 10.1016/j.postharvbio.2020.111231.

[ece372544-bib-0022] Cocucci, A. A. 1991. “Pollination Biology of *Nierembergia* (Solanaceae).” Plant Systematics and Evolution 174, no. 1–2: 17–35. 10.1007/bf00937691.

[ece372544-bib-0023] Cosacov, A. , A. A. Cocucci , and A. N. Sérsic . 2014. “Geographical Differentiation in Floral Traits Across the Distribution Range of the Patagonian Oil‐Secreting *Calceolaria polyrhiza*: Do Pollinators Matter?” Annals of Botany 113, no. 2: 251–266. 10.1093/aob/mct239. 24252281 PMC3890392

[ece372544-bib-0024] Cruz‐Valderrama, J. E. , J. J. Bernal‐Gallardo , H. Herrera‐Ubaldo , and S. de Folter . 2021. “Building a Flower: The Influence of Cell Wall Composition on Flower Development and Reproduction.” Genes 12, no. 7: 978. 10.3390/genes12070978. 34206830 PMC8304806

[ece372544-bib-0025] Davis, C. C. , C. D. Bell , S. Mathews , and M. J. Donoghue . 2002. “Laurasian Migration Explains Gondwanan Disjunction: Evidence From Malpighiaceae.” Proceedings of the National Academy of Sciences of the United States of America 99, no. 10: 6833–6837. 10.1073/pnas.10217589. 11983870 PMC124489

[ece372544-bib-0027] Dobson, H. E. 1987. “Role of Flower and Pollen Aromas in Host‐Plant Recognition by Solitary Bees.” Oecologia 2, no. 4: 618–623. 10.1007/BF00378991. 28312527

[ece372544-bib-0026] Dobson, H. E. , and G. Bergström . 2000. “The Ecology and Evolution of Pollen Odors.” Plant Systematics and Evolution 222, no. 1–4: 63–87. 10.1007/BF00984096.

[ece372544-bib-0028] Dötterl, S. , U. Füssel , A. Jürgens , and G. Aas . 2005. “1,4‐Dimethoxybenzene, a Floral Scent Compound in Willows That Attracts an Oligolectic Bee.” Journal of Chemical Ecology 31, no. 12: 2993–2998. 10.1007/s10886-005-9152-y. 16258713

[ece372544-bib-0029] Dötterl, S. , and I. Schäffler . 2007. “Flower Scent of Floral Oil‐Producing *Lysimachia punctata* as Attractant for the Oil‐Bee *Macropis fulvipes* .” Journal of Chemical Ecology 33, no. 2: 441–445. 10.1007/s10886-006-9237-2. 17151908

[ece372544-bib-0030] Dötterl, S. , and N. J. Vereecken . 2010. “The Chemical Ecology and Evolution of Bee–Flower Interactions: A Review and Perspectives.” Canadian Journal of Zoology 88, no. 7: 668–697. 10.1139/Z10-031.

[ece372544-bib-0031] Eckhardt, M. , M. Haider , S. Dorn , and A. Müller . 2014. “Pollen Mixing in Pollen Generalist Solitary Bees: A Possible Strategy to Complement or Mitigate Unfavourable Pollen Properties?” Journal of Animal Ecology 83, no. 3: 588–597. 10.1111/1365-2656.12168. 24164651

[ece372544-bib-0032] Edge, A. A. , B. N. van Nest , J. N. Johnson , et al. 2012. “Diel Nectar Secretion Rhythm in Squash (*Cucurbita pepo*) and Its Relation With Pollinator Activity.” Apidologie 43, no. 1: 1–16. 10.1007/s13592-011-0087-8.

[ece372544-bib-0033] Fenster, C. B. , W. S. Armbruster , P. Wilson , M. R. Dudash , and J. D. Thomson . 2004. “Pollination Syndromes and Floral Specialization.” Annual Review of Ecology, Evolution, and Systematics 35: 375–403. 10.1146/annurev.ecolsys.34.011802.132347.

[ece372544-bib-0034] Futuyma, D. J. , and A. A. Agrawal . 2009. “Macroevolution and the Biological Diversity of Plants and Herbivores.” Proceedings of the National Academy of Sciences of the United States of America 106, no. 43: 18054–18061. 19815508 10.1073/pnas.0904106106 PMC2775342

[ece372544-bib-0035] Gere, J. , and S. Timoshenko . 1997. Mechanics of Materials, 549. PWS.

[ece372544-bib-0108] Glas, J. J. , B. C. J. Schimmel , J. M. Alba , R. Escobar‐Bravo , R. C. Schuurink , and M. R. Kant . 2012. “Plant Glandular Trichomes as Targets for Breeding or Engineering of Resistance to Herbivores.” International Journal of Molecular Sciences 13, no. 12: 17077–17103. 10.3390/ijms131217077. 23235331 PMC3546740

[ece372544-bib-0036] Guimarães, M. M. , C. S. Souza , M. R. Sigrist , K. B. M. Miliato , and F. R. Maia . 2021. “Assessment of Interactions Between Oil Flowers and Floral Visitors in World Biomes.” Biological Journal of the Linnean Society 134, no. 2: 366–380. 10.1093/biolinnean/blab078.

[ece372544-bib-0109] Hancock, J. , S. J. Livingston , and L. Samuels . 2024. “Building a Biofactory: Constructing Glandular Trichomes in *Cannabis sativa* .” Current Opinion in Plant Biology 80: 102549. 10.1016/j.pbi.2024.102549. 38761520

[ece372544-bib-0037] Hermann, K. , and C. Kuhlemeier . 2011. “The Genetic Architecture of Natural Variation in Flower Morphology.” Current Opinion in Plant Biology 14, no. 1: 60–65. 10.1016/j.pbi.2010.09.012. 20934369

[ece372544-bib-0038] Homburger, J. , M. Pineirua , J. Casas , T. Speck , and F. Gallenmüller . 2025. “Within and Between‐Leg Oil Transfer in an Oil Bee.” Integrative Organismal Biology 7, no. 1: 1–14. 10.1093/iob/obaf025. PMC12215659 40606674

[ece372544-bib-0110] Johnson, S. D. , and K. E. Steiner . 2003. “Specialized Pollination Systems in Southern Africa.” South African Journal of Science 99: 345–348.

[ece372544-bib-0111] Kahnt, B. , W. N. Hattingh , P. Theodorou , et al. 2019. “Should I Stay or Should I Go? Pollinator Shifts Rather Than Cospeciation Dominate the Evolutionary History of South African *Rediviva* Bees and Their *Diascia* Host Plants.” Molecular Ecology 28, no. 17: 4118–4133. 10.1111/mec.15154. 31232488

[ece372544-bib-0039] Kaletnik, G. M. , M. G. Chausov , V. M. Shvayko , and V. M. Pryshlyak . 2011. Fundamentals of Engineering Methods of Strength and Stiffness Calculations, 616. Height‐Tech Press.

[ece372544-bib-0040] Kehrberger, S. , and A. Holzschuh . 2019. “How Does Timing of Flowering Affect Competition for Pollinators, Flower Visitation and Seed Set in an Early Spring Grassland Plant?” Scientific Reports 9: 15593. 10.1038/s41598-019-51916-0. 31666567 PMC6821694

[ece372544-bib-0112] Klumpers, S. G. T. , M. Stang , and P. G. L. Klinkhamer . 2019. “Foraging Efficiency and Size Matching in a Plant–Pollinator Community: The Importance of Sugar Content and Tongue Length.” Ecology Letters 22: 469–479. 10.1111/ele.13204. 30609161 PMC6850310

[ece372544-bib-0041] Kuhlmann, M. , and H. Hollens . 2014. “Morphology of Oil‐Collecting Pilosity of Female *Rediviva* Bees (Hymenoptera: Apoidea: Melittidae) Reflects Host Plant Use.” Journal of Natural History 49, no. 9–10: 561–573.

[ece372544-bib-0042] Kuhlmann, M. 2014. “Nest Architecture and Use of Floral Oil in the Oil‐Collecting South African Solitary Bee *Rediviva intermixta* (Cockerell) (Hymenoptera: Apoidea: Melittidae).” Journal of Natural History 48, no. 43–44: 2633–2644. 10.1080/00222933.2014.909069.

[ece372544-bib-0043] Lechantre, A. , A. Draux , H.‐A. B. Hua , D. Michez , P. Damman , and F. Brau . 2021. “Essential Role of Papillae Flexibility in Nectar Capture by Bees.” Proceedings of the National Academy of Sciences of the United States of America 118, no. 19: e2025513118. 33931548 10.1073/pnas.2025513118 PMC8126835

[ece372544-bib-0044] Lunau, K. 1996. “Signalling Functions of Floral Colour Patterns for Insect Flower Visitors.” Zoologischer Anzeiger 235, no. 1: 11–30.

[ece372544-bib-0114] MacGregor, G. R. , G. P. Nolan , S. Fiering , M. Roederer , and L. A. Herzenberg . 1991. “Chapter 17. Use of *E. coli* lacZ (β‐Galactosidase) as a Reporter Gene.” In Gene Transfer and Expression Protocols, edited by E. J. Murray , vol. 7, 217–235. *Methods in Molecular Biology* . 10.1385/0-89603-178-0:217. 21416358

[ece372544-bib-0113] Machado, I. C. , S. Vogel , and A. V. Lopes . 2002. “Pollination of Angelonia cornigera Hook. (Scrophulariaceae) by Long‐Legged, Oil‐Collecting Bees in NE Brazil.” Plant Biology 4, no. 3: 352–359. 10.1055/s-2002-32325.

[ece372544-bib-0045] Martins, A. C. , A. J. C. Aguiar , and I. Alves‐dos‐Santos . 2013. “Interaction Between Oil‐Collecting Bees and Seven Species of Plantaginaceae.” Flora 208, no. 7: 401–411. 10.1016/j.flora.2013.07.001.

[ece372544-bib-0046] Martins, A. C. , G. A. R. Melo , and S. S. Renner . 2015. “Gain and Loss of Specialization in Two Oil‐Bee Lineages, *Centris* and *Epicharis* (Apidae).” Evolution 69, no. 7: 1835–1844. 10.1111/evo.12689. 26095075

[ece372544-bib-0047] McManus, J. F. A. 1948. “Histological and Histochemical Uses of Periodic Acid.” Stain Technology 23, no. 3: 99–108. 10.3109/10520294809106232. 18867618

[ece372544-bib-0048] Michez, D. , A. Nel , J. J. Menier , and P. Rasmont . 2007. “The Oldest Fossil of a Melittid Bee (Hymenoptera: Apiformes) From the Early Eocene of Oise (France).” Zoological Journal of the Linnean Society 150, no. 4: 701–709. 10.1111/j.1096-3642.2007.00307.x.

[ece372544-bib-0050] Michez, D. , and S. Patiny . 2005. “World Revision of the Oil‐Collecting Bee Genus *Macropis* Panzer 1809 (Hymenoptera: Apoidea: Melittidae) With a Description of a New Species From Laos.” Annales de la Societe Entomologique de France 41, no. 1: 15–28. 10.1080/00379271.2005.10697439.

[ece372544-bib-0049] Michez, D. , S. Patiny , P. Rasmont , K. Timmermann , and N. J. Vereecken . 2008. “Phylogeny and Hostplant Evolution in Melittidae s.l. (Hymenoptera: Apoidea).” Apidologie 39, no. 1: 146–162. 10.1051/apido:2007048.

[ece372544-bib-0051] Michez, D. , M. Vanderplanck , and M. S. Engel . 2012. “Fossil Bees and Their Plant Associates.” In Evolution of Plant–Pollinator Relationships, edited by S. Patiny , 103–164. Cambridge University Press. 10.1017/CBO9781139014113.006.

[ece372544-bib-0115] Millett, F. , J. Standish , J. Scanley , et al. 2025. “The Fire Blight Pathogen *Erwinia amylovora* Enters Apple Leaves Through Naturally Occurring Wounds from the Abscission of Trichomes.” Plant Journal 123, no. 5: e70472. 10.1111/tpj.70472. 40944632

[ece372544-bib-0052] Neff, J. L. , and B. B. Simpson . 1981. “Oil‐Collecting Structures in the Anthophoridae (Hymenoptera): Morphology, Function, and Use in Systematics.” Journal of the Kansas Entomological Society 54, no. 1: 95–123.

[ece372544-bib-0053] Neff, J. L. , and B. B. Simpson . 2017. “Vogel's Great Legacy: The Oil Flower and Oil‐Collecting Bee Syndrome.” Flora 232: 104–116. 10.1016/j.flora.2017.01.003.

[ece372544-bib-0116] O'Donoghue, E. M. , S. D. Somerfield , L. M. Watson , et al. 2009. “Galactose Metabolism in Cell Walls of Opening and Senescing Petunia Petals.” Planta 229, no. 3: 709–721. 10.1007/s00425-008-0862-6. 19082620

[ece372544-bib-0054] Oliveira, L. C. , C. E. P. Nunes , V. L. G. Brito , and A. P. S. Caetano . 2022. “Floral Oil Production in a Family Dominated by Pollen Flowers: The Case of *Macairea radula* (Melastomataceae).” Flora 288: 152008. 10.1016/j.flora.2022.152008.

[ece372544-bib-0055] Ollerton, J. 1999. “The Evolution of Pollinator‐Plant Relationships Within the Arthropods.” In Evolution and Phylogeny of the Arthropoda, edited by A. Melic , J. J. DeHaro , M. Mendez , and I. Ribera , 741–758. Entomological Society of Aragon.

[ece372544-bib-0056] Ollerton, J. , R. Winfree , and S. Tarrant . 2011. “How Many Flowering Plants Are Pollinated by Animals?” Oikos 120, no. 3: 321–326. 10.1111/j.1600-0706.2010.18644.x.

[ece372544-bib-0057] Pauw, A. , B. Kahnt , M. Kuhlmann , et al. 2017. “Long‐Legged Bees Make Adaptive Leaps: Linking Adaptation to Coevolution in a Plant–Pollinator Network.” Proceedings of the Royal Society B: Biological Sciences 284: 20171707. 10.1098/rspb.2017.1707 PMC5597846 28904147

[ece372544-bib-0058] Pekkarinen, A. , Ø. Berg , I. Calabuig , L.‐Å. Janzon , and J. Luig . 2003. “Distribution and Co‐Existence of the *Macropis* Species and Their Cleptoparasite *Epeoloides coecutiens* (Fabr.) in NW Europe (Hymenoptera: Apoidea, Melittidae and Apidae).” Entomologica Fennica 14, no. 1: 53–59. 10.33338/ef.84171.

[ece372544-bib-0059] Peris, D. , J. Ollerton , H. Sauquet , et al. 2025. “Evolutionary Implications of a Deep‐Time Perspective on Insect Pollination.” Biological Reviews 100, no. 4: 1452–1466. 10.1111/brv.70008. 40070008 PMC12227787

[ece372544-bib-0060] Pesenko, Y. A. , V. G. Radchenko , and M. S. Kaygorodova . 1980. “The Ecology of Pollination of *Strigosella grandiflora* and *Erysimum badghysi* (Brassicaceae) by Wild Bees (Hymenoptera, Apoidea) in Badghys: Estimation of the Pressure of Competitive Relationships.” Entomological Review 59, no. 4: 58–73.

[ece372544-bib-0061] Pesenko, Y. A. , and V. G. Radchenko . 1993. “The Use of Bees (Hymenoptera, Apoidea) for Alfalfa Pollination: Main Directions and Modes, Methods for Evaluation of Populations of Wild Bees and Their Pollination Efficiency.” Entomological Review 72, no. 2: 101–119.

[ece372544-bib-0117] Policarová, J. , S. Cardinal , A. C. Martins , and J. Straka . 2019. “The Role of Floral Oils in the Evolution of Apid Bees (Hymenoptera: Apidae).” Biological Journal of the Linnean Society 128, no. 2: 486–497. 10.1093/biolinnean/blz099.

[ece372544-bib-0062] Portman, Z. M. , and V. J. Tepedino . 2017. “Convergent Evolution of Pollen Transport Mode in Two Distantly Related Bee Genera (Hymenoptera: Andrenidae and Melittidae).” Apidologie 48: 461–472. 10.1007/s13592-016-0489-8.

[ece372544-bib-0063] Possobom, C. C. F. , and S. R. Machado . 2018. “Elaiophores in Three Neotropical Malpighiaceae Species: A Comparative Study.” Plant Systematics and Evolution 304: 15–32. 10.1007/s00606-017-1443-6.

[ece372544-bib-0118] Pyke, G. H. 2016. “Plant‐pollinator Co‐evolution: It's Time to Reconnect with Optimal Foraging Theory and Evolutionarily Stable Strategies.” Perspectives in Plant Ecology, Evolution and Systematics 19: 70–76. 10.1016/j.ppees.2016.02.004.

[ece372544-bib-0064] Radchenko, V. G. 1996. “Evolution of Nest Building in Bees (Hymenoptera, Apoidea).” Entomological Review 75, no. 6: 20–32.

[ece372544-bib-0066] Radchenko, V. G. , Y. A. Pesenko , N. Y. Malysheva , and V. G. Veselovskiy . 1993. “Some Ways of Improving the Fecundity of the Alfalfa Leaf‐Cutter Bee Under Rearing Conditions.” Vestnik Zoologii 27, no. 5: 75–82.

[ece372544-bib-0065] Radchenko, V. G. , and Y. A. Pesenko . 1994. Biology of Bees (Hymenoptera, Apoidea), 350. Zoological Institute of the Russian Academy of Sciences. 10.13140/2.1.3938.6242.

[ece372544-bib-0067] Ramírez, S. R. , B. Gravendeel , R. B. Singer , C. R. Marshall , and N. E. Pierce . 2007. “Dating the Origin of the Orchidaceae From a Fossil Orchid With Its Pollinator.” Nature 448, no. 7157: 1042–1045. 10.1038/nature06039. 17728756

[ece372544-bib-0068] Rasmussen, C. , M. S. Engel , and N. J. Vereecken . 2020. “A Primer of Host‐Plant Specialization in Bees.” Emerging Topics in Life Sciences 4: 7–17. 10.1042/ETLS20190118. 32558903

[ece372544-bib-0069] Rasmussen, C. , and J. M. Olesen . 2000. “Oil Flowers and Oil‐Collecting Bees.” Det Norske Videnskaps‐Akademi. I 39: 23–31.

[ece372544-bib-0070] Renner, S. S. , and H. Schaefer . 2010. “The Evolution and Loss of Oil‐Offering Flowers: New Insights From Dated Phylogenies for Angiosperms and Bees.” Philosophical Transactions of the Royal Society, B: Biological Sciences 365, no. 1539: 423–435. 10.1098/rstb.2009.0229. PMC2838259 20047869

[ece372544-bib-0071] Rhizopoulou, S. , E. Spanakis , and A. Argiropoulos . 2015. “Study of Petal Topography of *Lysimachia arvensis* Grown Under Natural Conditions.” Acta Botanica Gallica 162, no. 4: 355–364. 10.1080/12538078.2015.1091985.

[ece372544-bib-0072] Rivest, S. , and J. R. Forrest . 2020. “Defence Compounds in Pollen: Why Do They Occur and How Do They Affect the Ecology and Evolution of Bees?” New Phytologist 225, no. 3: 1053–1064. 10.1111/nph.16230. 31569278

[ece372544-bib-0073] Roberts, R. B. , and S. R. Vallespir . 1978. “Specialization of Hairs Bearing Pollen and Oil on the Legs of Bees (Apoidea: Hymenoptera).” Annals of the Entomological Society of America 71, no. 4: 619–627. 10.1093/aesa/71.4.619.

[ece372544-bib-0074] Roulston, T. H. , J. H. Cane , and S. L. Buchmann . 2000. “What Governs Protein Content of Pollen: Pollinator Preferences, Pollen–Pistil Interactions, or Phylogeny?” Ecological Monographs 70, no. 4: 617–643. 10.1890/0012-9615(2000)070[0617:WGPCOP]2.0.CO;2.

[ece372544-bib-0075] Sasidharan, R. , R. R. Junker , E. J. Eilers , and C. Müller . 2023. “Floral Volatiles Evoke Partially Similar Responses in Both Florivores and Pollinators and Are Correlated With Non‐Volatile Reward Chemicals.” Annals of Botany 132, no. 1: 1–14. 10.1093/aob/mcad064. 37220889 PMC10550281

[ece372544-bib-0076] Schaefer, H. , and S. S. Renner . 2008. “A Phylogeny of the Oil Bee Tribe Ctenoplectrini (Hymenoptera: Anthophila) Based on Mitochondrial and Nuclear Data: Evidence for Early Eocene Divergence and Repeated Out‐of‐Africa Dispersal.” Molecular Phylogenetics and Evolution 47, no. 2: 799–811. 10.1016/j.ympev.2008.01.030. 18353689

[ece372544-bib-0077] Schäffler, I. , F. Balao , and S. Dötterl . 2012. “Floral and Vegetative Cues in Oil‐Secreting and Non‐Oil‐Secreting *Lysimachia* Species.” Annals of Botany 110, no. 1: 125–138. 10.1093/aob/mcs101. 22634256 PMC3380597

[ece372544-bib-0078] Schäffler, I. , and S. Dötterl . 2011. “A Day in the Life of an Oil Bee: Phenology, Nesting, and Foraging Behavior.” Apidologie 42, no. 3: 409–424. 10.1007/s13592-011-0010-3.

[ece372544-bib-0079] Scheller, H. V. , and P. Ulvskov . 2010. “Hemicelluloses.” Annual Review of Plant Biology 61: 263–289. 10.1146/annurev-arplant-042809-112315. 20192742

[ece372544-bib-0080] Schiestl, F. P. , and R. Peakall . 2005. “Two Orchids Attract Different Pollinators With the Same Floral Odour Compound: Ecological and Evolutionary Implications.” Functional Ecology 19, no. 4: 674–680. 10.1111/j.1365-2435.2005.01010.x.

[ece372544-bib-0081] Shimizu, A. , I. Dohzono , M. Nakaji , et al. 2014. “Fine‐Tuned Bee‐Flower Coevolutionary State Hidden Within Multiple Pollination Interactions.” Scientific Reports 4: 3988. 10.1038/srep03988. 24496444 PMC3913927

[ece372544-bib-0082] Simpson, B. B. , and J. L. Neff . 1981. “Floral Rewards: Alternatives to Pollen and Nectar.” Annals of the Missouri Botanical Garden 68, no. 2: 301–322. 10.2307/2398800.

[ece372544-bib-0083] Simpson, B. B. , J. L. Neff , and D. Seigler . 1977. “ *Krameria*, Free Fatty Acids and Oil‐Collecting Bees.” Nature 267, no. 5607: 150–151. 10.1038/267150a0. 16073425

[ece372544-bib-0084] Simpson, B. B. , J. L. Neff , and D. S. Seigler . 1983. “Floral Biology and Floral Rewards of *Lysimachia* (Primulaceae).” American Midland Naturalist 110, no. 2: 249–256. 10.2307/2425266.

[ece372544-bib-0119] Stang, M. , P. G. L. Klinkhamer , N. M. Waser , I. Stang , and E. van der Meijden . 2009. “Size‐Specific Interaction Patterns and Size Matching in a Plant–Pollinator Interaction Web.” Repeated Measures Annals of Botany 103, no. 9: 1459–1469. 10.1093/aob/mcp027. PMC2701768 19228701

[ece372544-bib-0120] Steiner, K. E. , and V. B. Whitehead . 1988. “The Association Between Oil‐Producing Flowers and Oil‐Collecting Bees in the Drakensberg of Southern Africa.” In Modern Systematic Studies in African Botany, edited by P. Goldblatt and P. P. Lowry , vol. 25, 259–277. Monographs in Systematic Botany of the Missouri Botanical Garden.

[ece372544-bib-0121] Steiner, K. E. , and V. B. Whitehead . 1990. “Pollinator Adaptation to Oil‐Secreting Flowers *Rediviva* and *Diascia* .” Evolution 44, no. 6: 1701–1707. 10.1111/j.1558-5646.1990.tb03857.x. 28564320

[ece372544-bib-0088] Steiner, K. E. , and V. B. Whitehead . 1990. “Pollinator Adaptation to Oil‐Secreting Flowers–*Rediviva* and *Diascia* .” Evolution 44: 1701–1707. 28564320 10.1111/j.1558-5646.1990.tb03857.x

[ece372544-bib-0086] Steiner, K. E. , and V. B. Whitehead . 1991. “Oil Flowers and Oil Bees: Further Evidence for Pollinator Adaptation.” Evolution 45: 1493–1501. 10.1111/j.1558-5646.1991.tb02651.x. 28563824

[ece372544-bib-0087] Steiner, K. E. , and V. B. Whitehead . 2002. “Oil Secretion and the Pollination of *Colpias mollis* (Scrophulariaceae).” Plant Systematics and Evolution 235, no. 1: 53–66. 10.1007/s00606-002-0216-y.

[ece372544-bib-0090] Thorp, R. W. 1979. “Structural, Behavioral, and Physiological Adaptations of Bees (Apoidea) for Collecting Pollen.” Annals of the Missouri Botanical Garden 66, no. 4: 788–812. 10.2307/2398919.

[ece372544-bib-0089] Thorp, R. W. 2000. “The Collection of Pollen by Bees.” Plant Systematics and Evolution 222, no. 1–4: 211–223. 10.1007/BF00984103.

[ece372544-bib-0091] Tong, Z.‐Y. , L.‐Y. Wu , H.‐H. Feng , et al. 2023. “New Calculations Indicate That 90% of Flowering Plant Species Are Animal‐Pollinated.” National Science Review 10, no. 10: nwad219. 10.1093/nsr/nwad219. 37743955 PMC10517183

[ece372544-bib-0124] Triponez, Y. , N. Arrigo , A. Espíndola , and N. Alvarez . 2015. “Decoupled Post‐Glacial History in Mutualistic Plant–Insect Interactions: Insights from the Yellow Loosestrife (*Lysimachia vulgaris*) and Its Associated Oil‐Collecting Bees (*Macropis europaea and M. fulvipes*).” Journal of Biogeography 42, no. 4: 630–640. 10.1111/jbi.12456.

[ece372544-bib-0092] Van der Kooi, C. J. , and J. Ollerton . 2020. “The Origins of Flowering Plants and Pollinators.” Science 368, no. 6497: 1306–1308. 10.1126/science.aay3662. 32554579

[ece372544-bib-0093] Van der Niet, T. , and S. D. Johnson . 2012. “Phylogenetic Evidence for Pollinator‐Driven Diversification of Angiosperms.” Trends in Ecology & Evolution 27, no. 6: 353–361. 10.1016/j.tree.2012.02.002. 22445687

[ece372544-bib-0094] Vanderplanck, M. , N. J. Vereecken , L. Grumiau , et al. 2017. “The Importance of Pollen Chemistry in Evolutionary Host Shifts of Bees.” Scientific Reports 7: 43058. 10.1038/srep43058. 28216663 PMC5316986

[ece372544-bib-0095] Vaudo, A. D. , E. Lin , J. A. Luthy , A. S. Leonard , and E. M. Grames . 2024. “Do Past and Present Abiotic Conditions Explain Variation in the Nutritional Quality of Wildflower Pollens for Bees?” Evolutionary Ecology 38, no. 4: 941–955. 10.1007/s10682-024-10313-4.

[ece372544-bib-0096] Vaudo, A. D. , J. F. Tooker , C. M. Grozinger , and H. M. Patch . 2015. “Bee Nutrition and Floral Resource Restoration.” Current Opinion in Insect Science 10: 133–141. 10.1016/j.cois.2015.05.008. 29588000

[ece372544-bib-0103] Vogel, S. 1971. “Ölproduzierende Blumen, die Durch Ölsammelnde Bienen Bestäubt Werden.” Naturwissenschaften 58: 58. 10.1007/BF00620817.5543097

[ece372544-bib-0101] Vogel, S. 1974. “Ölblumen und Ölsammelnde Bienen.” Tropische Und Subtropische Pflanzenwelt 7: 285–547.

[ece372544-bib-0098] Vogel, S. 1984. “The *Diascia* Flower and Its Bee—An Oil‐Based Symbiosis in Southern Africa.” Acta Botanica Neerlandica 33, no. 4: 509–518. 10.1111/j.1438-8677.1984.tb01842.x.

[ece372544-bib-0097] Vogel, S. , and A. Cocucci . 1995. “Pollination of *Basistemon* (Scrophulariaceae) by Oil‐Collecting Bees in Argentina.” Flora 190, no. 4: 353–363. 10.1016/S0367-2530(17)30677-1.

[ece372544-bib-0099] Vogel, S. , and I. C. Machado . 1991. “Pollination of Four Sympatric Species of *Angelonia* (Scrophulariaceae) by Oil‐Collecting Bees in NE, Brazil.” Plant Systematics and Evolution 178, no. 3: 153–178. 10.1007/BF00937962.

[ece372544-bib-0100] Vogel, S. , and C. D. Michener . 1985. “Long Bee Legs and Oil‐Producing Floral Spurs, and a New *Rediviva* (Hymenoptera, Melittidae; Scrophulariaceae).” Journal of the Kansas Entomological Society 58, no. 2: 359–364.

[ece372544-bib-0102] Vogel, S. 1986. “Ölblumen und Ölsammelnde Bienen. Zweite Folge. *Lysimachia* und *Macropis* .” Tropische Und Subtropische Pflanzenwelt 54: 147–312.

[ece372544-bib-0104] Weiner, C. N. , A. Hilpert , M. Werner , K. E. Linsenmair , and N. Blüthgen . 2010. “Pollen Amino Acids and Flower Specialisation in Solitary Bees.” Apidologie 41, no. 4: 476–487. 10.1051/apido/2009083.

[ece372544-bib-0122] Whitehead, V. B. , and N. C. Anthony . 1984. “The Bee, *Rediviva Longimanum* Michener (Apoidea: Melittidae), Collecting Pollen and Oil from *Diascia longicornis* (Thunb.) Druce (Scrophulariaceae).” South African Journal of Science 80: 286.

[ece372544-bib-0123] Zhao, Z. , O. E. Shklyaev , A. Nili , et al. 2013. “Cellulose Microfibril Twist, Mechanics, and Implication for Cellulose Biosynthesis.” Journal of Physical Chemistry A 117: 2580–2589. 10.1021/jp3089929. 23418823

